# Conserved roles of GATA4 and its target gene *TBX2* in regulation of human cardiogenesis

**DOI:** 10.1242/bio.062553

**Published:** 2026-06-15

**Authors:** Nicola Graham, Pavel Kirilenko, Ilya Patrushev, Ewan D. Fowler, Peter Kille, Michael Gilchrist, Nick D. L. Owens, Branko Latinkic

**Affiliations:** ^1^ School of Biosciences, Cardiff University, Cardiff CF10 3AX, UK; ^2^ The Francis Crick Institute, London NW1 1AT, UK; ^3^ Department of Clinical and Biomedical Sciences, Exeter University, Exeter EX2 5DW, UK

**Keywords:** Cardiogenesis, GATA4, *Xenopus*, Human iPSCs

## Abstract

The transcription factor GATA4 is a key mediator of cardiogenesis, regulating this process through the expression of downstream target genes, only a subset of which have been identified. Using a gain-of-function model based on pluripotent ectoderm explants from *Xenopus* embryos expressing GATA4, we identified a set of GATA4 targets that are also regulated by cardiogenic Nodal signalling. GATA4 was shown to be required for the expression of the target genes *tbx2* and *prdm1*, both of which were subsequently found to have roles of their own *in vivo*, as downregulation of *tbx2*, a positive target, and overexpression of *prdm1*, a negative target, interferes with cardiac development in *Xenopus* embryos. The conservation of the *GATA4-TBX2-PRDM1* regulatory relationship was demonstrated in human induced pluripotent stem cell-derived cardiomyocytes. Loss of GATA4 function resulted in downregulation of *TBX2*, upregulation of *PRDM1* and failure of cardiogenesis. GATA4-deficient cells failed to form normal cardiomyocytes, with most cells adopting alternative fates and only a small minority displaying an aberrant cardiomyocyte phenotype. Genome-wide transcriptomic analysis documented a marked reduction in cardiomyocyte and endothelial cell transcriptomes, accompanied by upregulation of transcriptional profiles associated with smooth muscle cells and fibroblasts. Disruption of *TBX2* led to the formation of cardiomyocytes with hypertrophic-like features consistent with maladaptive remodelling. In addition, although *PRDM1* was not essential for cardiomyocyte formation, it appeared to play a role in fine-tuning the timing and levels of gene expression. Together, these findings establish a conserved regulatory relationship between GATA4 and its target genes *TBX2* and *PRDM1* and suggest important roles for these factors in modulating cardiomyocyte development.

## Teaser

**Summary:** The regulatory relationship between cardiogenic factor GATA4 and its target genes *TBX2* and *PRDM1*, discovered in *Xenopus*, is conserved in human cardiogenesis.

## INTRODUCTION

Heart development and homeostasis are controlled by the collective action of numerous transcription factors (TFs), which come together to form complex and dynamic gene regulatory networks (GRNs) that define and maintain cellular identity. Disruption of the cardiac GRN is associated with the development of congenital heart defects (CHDs) and cardiovascular disease (CVD) in later life (Fahed et al., 2014; Tan et al., 2002). The established core network of TFs that orchestrate cardiac development includes GATA4/5/6, NKX2-5, HAND1, 2, TBX2/5/20 and proteins belonging to the MADS domain family such as MEF2 and SRF (Akerberg and Pu, 2020; Bruneau, 2013; Olson, 2006; Paige et al., 2015). Evidence from mouse knockout models has shown that the absence of any one of these factors results in an array of severe cardiac defects that almost invariably lead to embryonic lethality (Bruneau et al., 2001; Firulli et al., 1998; Koutsourakis et al., 1999; Kuo et al., 1997; Srivastava et al., 1997; Stennard et al., 2005; Watt et al., 2004), highlighting the vulnerability of cardiac GRNs to perturbation.

GATA4 is a core member of the cardiac GRN with well-documented roles in all stages of heart development as well as in postnatal life. Early experiments in mice identified an indirect role for GATA4 in visceral endoderm, which is essential for cardiac morphogenesis in the overlying mesoderm (Kuo et al., 1997; Molkentin et al., 1997). Subsequent studies have identified cell-autonomous roles for GATA4 in the developing heart (Watt et al., 2004; Zeisberg et al., 2005). For example, a heterozygous dominant mutation, G296S, was strongly associated with CHD, including a range of severe atrial and ventricular septal defects often requiring surgical correction (Garg et al., 2003). In contrast, heterozygous mice carrying the corresponding mutation developed only mild defects in postnatal atrial septal closure (Misra et al., 2012), highlighting the limitations of mouse models of CHD. Cardiomyocytes (CMs) generated from GATA4 (G296S) patient-derived induced pluripotent stem cells (iPSCs) show defects in contractility and metabolism (Ang et al., 2016). The molecular basis for this phenotype was shown to be the inability of the mutant GATA4 protein to recruit TBX5 to common targets in chromatin, leading to downregulation of cardiac targets and to aberrant de-repression of endothelial targets (Ang et al., 2016).

In addition to being required for heart development, GATA4 is sufficient to drive CM differentiation in permissive embryonic cells, either on its own in pluripotent ectodermal explants from *Xenopus* embryos (Latinkić et al., 2003) or together with Baf60c in mouse early embryonic mesoderm (Takeuchi and Bruneau, 2009). Furthermore, GATA4 is used in most cocktails of cardiac TFs for reprogramming of non-cardiac cells to CMs (Garry et al., 2022). For example, *GATA4*, along with additional cardiac GRN members *MEF2* and *TBX5*, is sufficient for reprogramming of fibroblasts to CMs (Garry et al., 2022; Xie et al., 2022). The GATA4 requirement for normal cardiac development, as outlined above, and the ability of GATA4 to steer the genomic programme of non-CMs towards a CM fate reaffirm it as a key component of the cardiac GRN.

Mechanistic studies focused on mapping of GATA4 occupancy sites in chromatin in models such as mouse embryos, human iPSC-derived CMs (iPSC-CMs) and adult hypertrophic hearts are beginning to identify the roles of GATA4 at the chromatin level by defining its stage-, signal- and cell-type-specific targets. Besides the core cardiac GRN, numerous additional TFs regulate multiple aspects of cardiac development such as cardiac cell type diversification and morphogenesis. The emerging expanded cardiac GRN includes genes such as *TBX2*, shown to regulate the formation of the atrioventricular canal (AVC) by repressing the transcriptional programme for chamber CMs. To describe an expanded cardiac GRN, it will be necessary to obtain a better knowledge of regulatory relationships between its nodes.

A simple model for studying the cardiac GRN is offered by overexpression of GATA4 in pluripotent ectodermal explants from blastula stage *Xenopus* embryos. GATA4 activity is sufficient to reprogram explant cells fated to become epidermis towards CMs and is enhanced further by Wnt antagonism in a process, which mimics normal heart development by producing beating cardiac tissue (Latinkić et al., 2003). In this model, GATA4 also induces endothelial cells as well as anterior endoderm, in keeping with its known roles *in vivo*.

Pluripotent explants from *Xenopus* embryos can also be directed towards CM fate by Nodal/Activin signalling, the principal inducers of mesoderm and endoderm in vertebrate embryos (Logan and Mohun, 1993; Takahashi et al., 2000). In *Xenopus* explants, Nodal signalling acts in a concentration-dependent manner, with a high dose required for induction of endoderm and CMs and a lower dose for skeletal muscle (Logan and Mohun, 1993; Takahashi et al., 2000).

We used both GATA4 and Nodal cardiogenic triggers to identify and characterise putative components of the cardiac GRNs as co-regulated differentially expressed genes (DEGs). A subset of DEGs was validated as GATA4 targets *in vivo* as well as in explants. Interference with the normal expression of two such targets, *tbx2* and *prdm1*, is shown to affect heart development in *Xenopus* embryos.

The wider relevance of the data obtained in *Xenopus* is demonstrated by identifying the essential roles of GATA4 and its target gene *TBX2* in the differentiation of human iPSC-CMs.

## RESULTS

### Identification of GATA4 targets under cardiogenic conditions

**Fig. 1.**
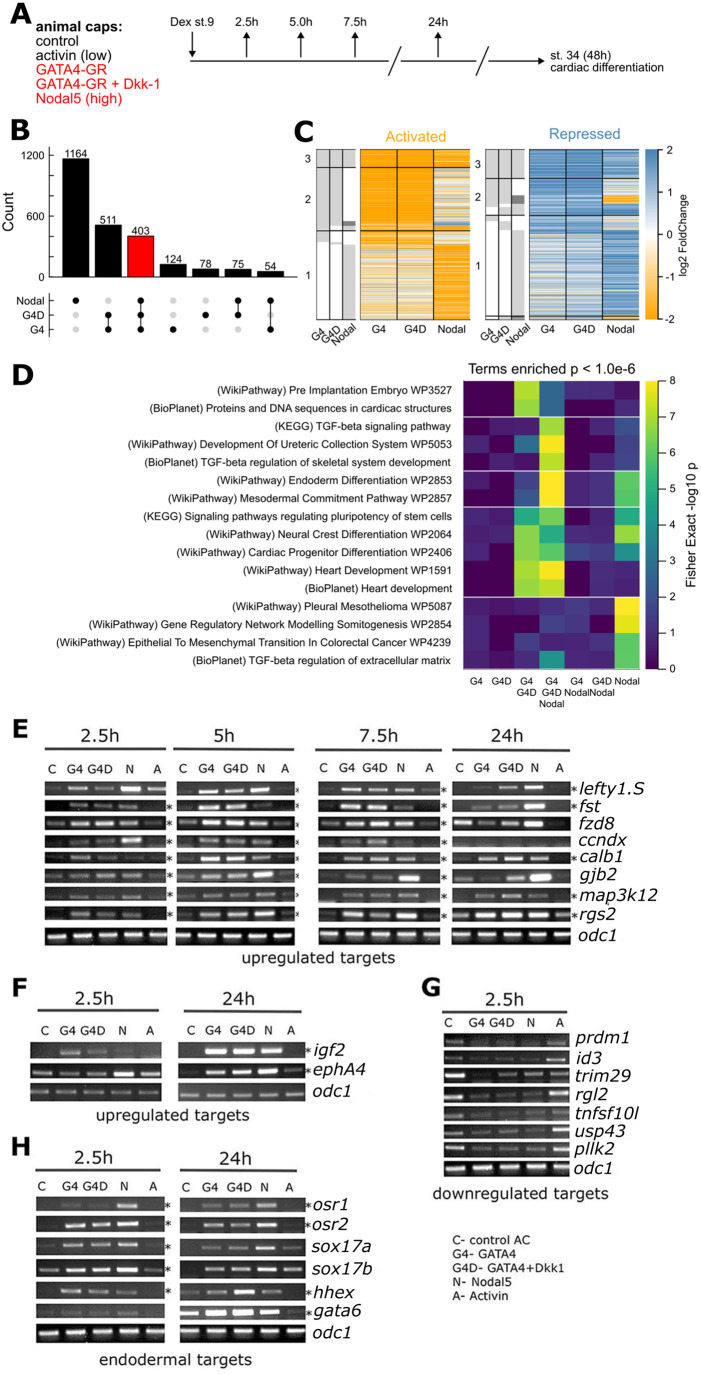
**Identification and validation of cardiogenesis-related targets of GATA4.** (A) Experimental outline. Animal cap explants from blastula-stage embryos were excised and incubated for the indicated time before being harvested and subjected to RNA-seq analysis. The samples injected with mRNAs coding for proteins known to induce cardiac tissue are indicated in red. GATA4-GR was activated by incubation in 1 µM DEX. The control samples were uninjected animal cap explants. Animal caps treated with a low dose of soluble activin, known to induce skeletal but not cardiac muscle (Fig. S1), were used as an additional control and a method to select genes responsive to cardiac-inducing conditions. st., stage. (B) UpSet plot describing intersection of DEGs irrespective of time point compared to control. Bars indicate the total number of genes differentially expressed in combination of conditions indicated in the lower plot at any time point. (C) A heatmap showing genes activated and repressed per condition irrespective of time point. Left grey heatmap indicates whether a gene was significantly differentially expressed in the indicated condition. A corresponding heatmap divided per time point is shown in Fig. S1E. (D) Gene set enrichment calculated using Enrichr. The heatmap shows −log10 FDR for enrichment of the indicated term for gene sets defined by the UpSet plot in B, within WikiPathways, BioPlanet and Kyoto Encyclopedia of Genes and Genomes (KEGG) annotations. The heatmap is divided into five classes of differing enrichment responses. Gene set enrichments for GO are provided in Fig. S2, and the table of all tested terms is provided in Table S1. (E,F) RT-PCR validation of a subset of upregulated DEGs. Time points where each DEG was validated are indicated by an asterisk. *odc1* expression is used as a loading control. The same *odc1* data are shown in all panels where the same cDNA samples were used (Fig. 1E,G,H, Fig. 2A,D; Fig. S3A). (G) RT-PCR validation of downregulated DEGs at 2.5 h of incubation. All DEGs were validated as downregulated by GATA4-GR, GATA4-GR+Dkk-1 and Nodal 5 compared to the level of expression in uninjected or Activin-treated animal caps. (H) RT-PCR validation of DEGs linked with endoderm development at 2.5 and 24 h. Time points where each DEG was validated are indicated by an asterisk.

To dissect the genomic programme for cardiogenesis regulated by GATA4, we employed a well-established gain-of-function model using pluripotent ectodermal explants from blastula-stage *Xenopus* embryos (Latinkić et al., 2003). As GATA4 plays key roles in the development of multiple cell types of mesodermal and endodermal origin and is sufficient to specify endodermal, endothelial and blood cells in *Xenopus* animal cap explants, we designed an experimental approach for the selection of cardiogenesis-related genes regulated by GATA4. This consisted of defining a set of genes regulated by cardiogenic conditions: GATA4 (G4); GATA4 in the presence of Wnt antagonist Dkk-1 (G4D), which enhances cardiogenesis; and high levels of Nodal/Activin signalling, known to induce cardiogenesis (Fig. 1; Fig. S1). At the same time, these genes were not regulated by a lower level of Nodal/Activin, which induces skeletal but not cardiac muscle and by Dkk-1 alone (Fig. 1; Fig. S1). In this model, it takes approximately 2 days for GATA4 to induce cardiac differentiation as assessed by the expression of CM markers (Fig. 1A; Fig. S1A).

**Fig. 2.**
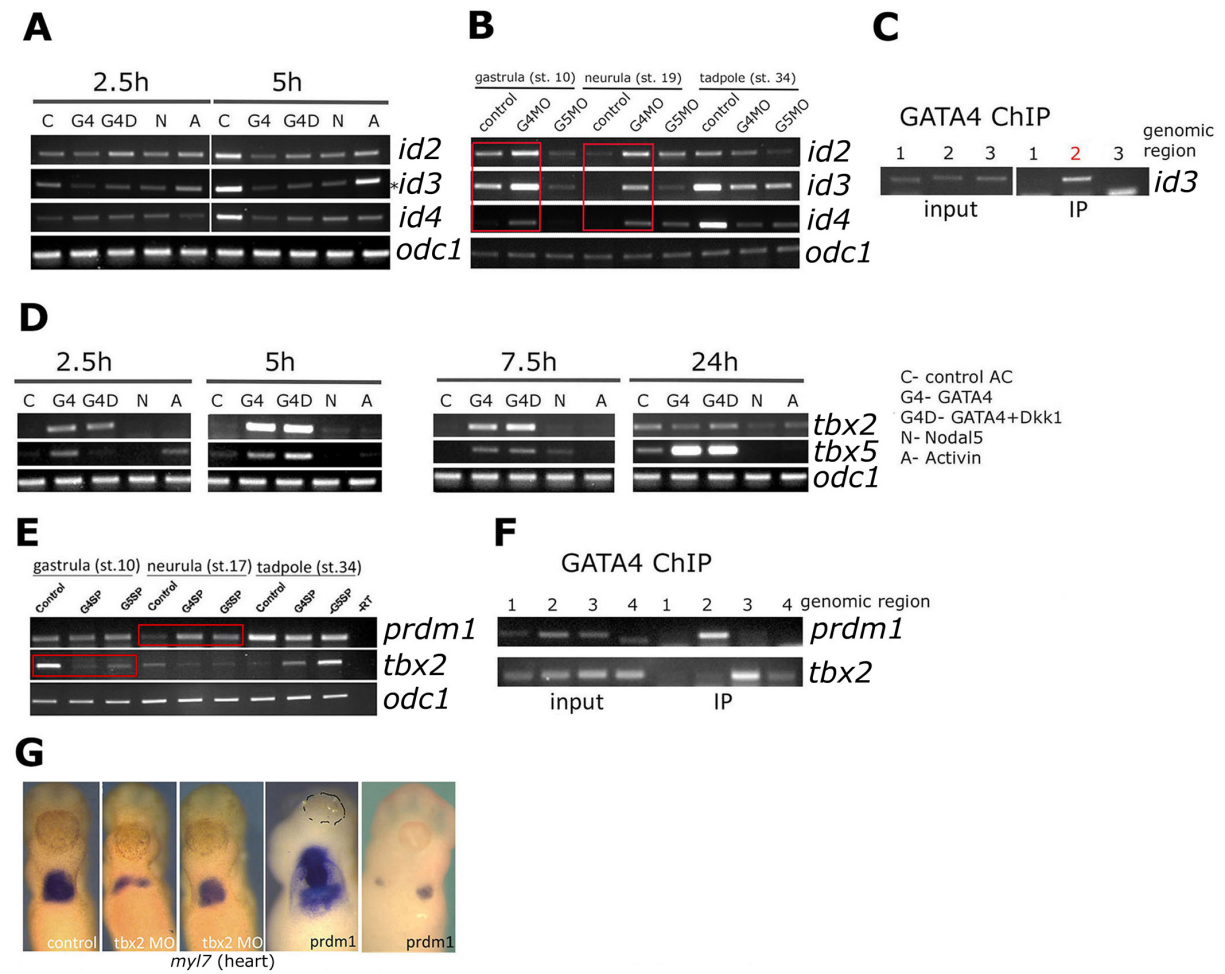
***id3*, *tbx2* and *prdm1* are regulated by GATA4.** (A) RT-PCR analysis for the expression of *id2-id4* genes in control (uninjected), GATA4-injected, GATA4+Dkk-1-injected, Nodal-5-injected and Activin-treated animal cap explants at 2.5 and 5 h after excision of animal cap explants and activation of inducible GATA4-GR by 1 µM DEX. *id3* is downregulated by cardiogenic conditions at 2.5 h and more strongly at 5 h. This contrasts with family members *id2* and *id4*, which were not picked up in the screen. *odc1* expression is used as the loading control. The same *odc1* data are shown in all panels where the same cDNA samples were used (Fig. 1E,G,H, Fig. 2A,D; Fig. S3A). (B) GATA4 is required for regulation of *id2-id4* gene expression *in vivo*. Embryos injected with control MOs, GATA4 MOs (G4MO) or GATA5 MOs (G5MO) were collected at indicated stages and analysed for expression of *id2-id4* and *odc1* by RT-PCR. Downregulation of GATA4 (G4MO samples) leads to upregulation of *id2-id4* expression at st. 10 and st. 19 (indicated by the red boxes), as assessed by RT-PCR. (C) GATA4 associates with the *id3* genomic locus. Three genomic regions containing GATA sites were amplified from genomic DNA (input) or from DNA immunoprecipitated (IP) by antibodies against HA-tagged GATA4. Region 2, but not 1 and 3, associates with GATA4. (D) Validation of *tbx2* and *tbx5* as GATA4 targets in animal cap explants. Samples collected at the indicated time points were analysed by RT-PCR for expression of *tbx2*, *tbx5* and *odc1*. *tbx2* is strongly induced by GATA4 and GATA4+Dkk-1 and weakly by Nodal 5 at 5 h; tbx5 is induced by all three cardiogenic conditions at 7.5 h and by GATA4 and GATA4+Dkk-1 at 5 and 24 h. (E) GATA4 is required for regulation of *prdm1* and *tbx2 in vivo*. *prdm1* expression in both GATA4 splice-blocking MO (G4SP) and GATA5 splice-blocking MO (G5SP) is upregulated compared to control MO-injected embryos at st. 17, whereas *tbx2* expression is downregulated upon GATA4 and GATA5 knockdown (indicated by red boxes). (F) GATA4 binds discrete genomic regions of *prdm1* and *tbx2*. Two additional GATA4-binding regions were identified for *prdm1* and are shown in Fig. S3B. Region 2 is the same as amplicon 12 in Fig. S3B. Sequences of all positive amplicons are shown in Fig. S3C. (G) Downregulation of *tbx2* and gain of function of *prdm1* lead to heart abnormalities *in vivo*. Ventral views of representative st. 34 embryos (anterior to the top) with the heart revealed by *myl7* WMISH. Injection of *tbx2* MO leads to cardia bifida or a smaller heart region. Injection of *prdm1* mRNA (‘prdm1’ in the figure) causes defective development of the heart (linear heart tube or cardia bifida). Additional examples and phenotype classification are shown in Fig. S3D,E. The dashed line outlines the cement gland in a sample in which it is not clear.

We focused on the early stages of the genomic programme, which was expected to include immediate-early and delayed-early targets, by sampling at 2.5, 5, 7.5 and 24 h after excision of animal cap explants and activation of inducible GATA4-GR fusion by addition of dexamethasone (DEX). We performed poly(A)^+^ RNA sequencing (RNA-seq) and calculated DEGs at each stage relative to control. The three conditions produced a highly concordant impact on gene expression for each time point (Fig. S1B-D); therefore, we combined these to define DEGs per condition. We found 1092 DEGs (690 activated and 402 repressed) for GATA4 (G4), 1067 (602 activated and 465 repressed) for GATA4+Dkk-1 (G4D), and 1696 (829 activated and 867 repressed) for Nodal. Comparing between conditions, we found exceptional overlap of 914 genes differentially expressed in G4 and G4D, 83.7% and 85.7% of their genes, respectively (Fig. 1B). The overlap with Nodal DEGs was more limited, with 403 genes shared between the three cardiogenic treatment groups (Fig. 1B). We performed gene set enrichment using Enrichr (Kuleshov et al., 2016) (Fig. 1D; Fig. S2, Table S1), and we calculated enrichments for all intersection groups indicated in the UpSet plot (Fig. 1B). We found a clear induction of cardiac gene expression programmes in the intersection of GATA4 and Nodal DEGs, with terms such as Heart Development [false discovery rate (FDR)<10^−7^, intersection of G4/G4D/Nodal] and Cardiac Progenitor Differentiation (FDR<10^−5^, intersection of G4/G4D/Nodal). We further found an enrichment in Gene Ontology (GO) terms related to TF-driven regulation of gene expression in the intersection of GATA4 and Nodal conditions, with enrichment of terms such as Regulation Of Transcription By RNA Polymerase II (FDR<10^−23^) (Fig. S2).

Validation confirmed DEGs that satisfied the selection criteria (regulated by G4, G4D and high Nodal but not low Nodal/Activin) at all time points such as *fst*, *map3k12*, and *rgs2* (Fig. 1E). Other DEGs were validated at some but not all time points.

Upregulated DEGs include the known Nodal target *lefty1* (Cheng et al., 2000); at 2.5 h, *lefty1.S* is strongly induced by Nodal, as expected from a direct target of Nodal signalling (Fig. 1E). However, as the gene is also induced by a low level of Nodal/Activin signalling at that time, it is not validated as a DEG induced by cardiogenic conditions. The expression of *lefty1.S* induced by cardiogenic but not non-cardiogenic conditions is sustained beyond 2.5 h, thus identifying it as a DEG (Fig. 1E). Additional upregulated DEGs include *igf2* and *ephA4* (Fig. 1F), Tbx family TFs *tbx2* and *tbx5* (Fig. 2D) and *gata2*, *hoxc13* and *lmo2* (Fig. S3).

In addition to upregulated DEGs, we have identified DEGs that are downregulated in response to cardiac-inducing conditions (Fig. 1G). They were most consistently observed at 2.5 h, the proximal time point at which a direct action of G4, G4D and Nodal is most likely. Downregulated DEGs include TFs *prdm1* and *id3*; signalling molecules such as *pllk2*, *tnfsf10l*, and *rgl2*; E3 ubiquitin ligase *trim29* and ubiquitin peptidase *usp43* (Fig. 1G). Since *id3* is a member of a small family of TFs (*id2-id4* in *Xenopus*) with described developmental roles, including in the heart (Cunningham et al., 2017), we have also examined the expression of *id2* and *id4* under the conditions of our screen. As shown in Fig. 2A, in contrast to *id3*, *id2* and *id4* are not found to be differentially expressed.

Whilst the screen conditions induced a programme of cardiogenesis, none of the three treatments were strictly cardiogenic. Indeed, our GO analysis has indicated enrichment of terms such as Endoderm Differentiation (Fig. 1D), and we found early endodermal genes among DEGs at 2.5 h and TFs *osr1*, *osr2*, *sox17a*, *sox17b* and *hhex* (Fig. 1H).

### Regulation of *tbx2*, *id3* and *prdm1* by GATA4

To validate identified DEGs as physiologically relevant targets of GATA4, we examined their regulation by GATA4 *in vivo*. As shown in Fig. 2B, downregulation of GATA4 using morpholino oligonucleotides (MOs) leads to upregulation of *id3* at gastrula and neurula stages; interestingly, even though *id2* and *id4* were not confirmed as DEGs, their expression was also upregulated by GATA4 MOs, suggesting an indirect mode of action. Downregulation of GATA4 and the most closely related cardiogenic GATA factor, GATA5, led to upregulation of *prdm1* and to downregulation of *tbx2* (Fig. 2E). This analysis has shown that GATA4 is required for the normal expression of *id3*, *prdm1* and *tbx2 in vivo*.

We next asked if GATA4 regulates *id3*, *prdm1* and *tbx2* through association with their regulatory regions. For this analysis, we identified consensus GATA-binding sites within 10 kb upstream and 2 kb downstream of their translation start sites and tested their presence in genomic DNA associated with exogenous GATA4. For all three genes, the minority of tested sites was found to associate with GATA4 (Fig. 2C,F; Fig. S3B,C), suggesting that GATA4 is directly and specifically regulating their expression.

We subsequently tested whether *tbx2* and *prdm1* have roles of their own in *Xenopus* heart development. The expression of *tbx2*, a GATA4 target upregulated by cardiogenic conditions, was downregulated by MOs, and this led to reduced and deformed heart formation in a majority of embryos (Fig. 2G; Fig. S3D). The role of *prdm1* as a target of GATA4-mediated downregulation was tested by mRNA-mediated overexpression, which caused morphological defects such as cardia bifida and linear heart tube (Fig. 2G; Fig. S3E). These results indicate that *tbx2* and *prdm1* are targets of GATA4 in cardiogenesis and that their normal expression is required for early heart development in *Xenopus* embryos.

### GATA4 is required for the normal expression of *PRDM1* and *TBX2* during human iPSC-CM differentiation

**Fig. 3.**
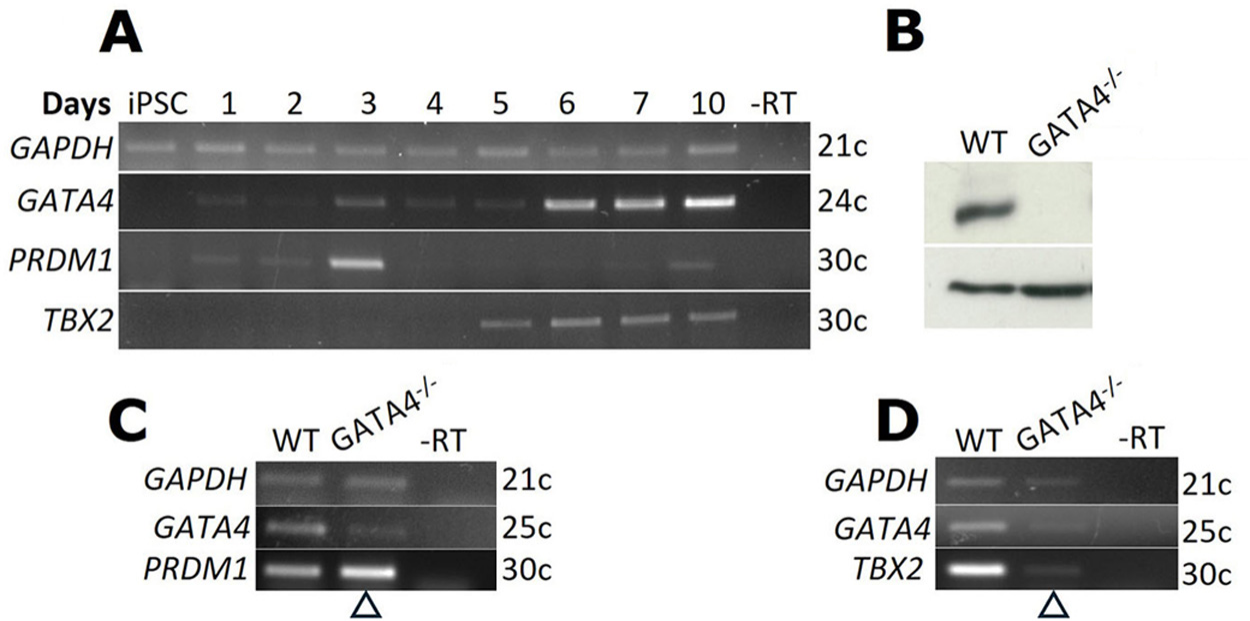
**GATA4 is required for regulation of its target genes *PRDM1* and *TBX2* in iPSC-CMs.** (A) Analysis of the expression of GATA4 and its target genes during iPSC-CM differentiation was examined using RT-PCR. Representative of three biological repeats. (B) A western blot showing the absence of GATA4 protein at day 4 in the GATA4-null lines created using CRISPR-Cas9 gene editing. Representative of three biological repeats. ERK2 is used as a loading control. (C) RT-PCR data showing the expression of *GATA4* in mutant cells at day 4 is reduced versus an increase in the expression of *PRDM1* (arrowhead) when compared to WT cells. (D) RT-PCR for day 10 samples showing *GATA4* expression remains lower in the mutant line, as is *TBX2* (arrowhead). For all RT-PCRs, *GAPDH* has been used as a normalisation control. RT-PCRs in C and D are representative of two biological repeats.

We next sought to establish whether these findings are translatable to human development using iPSC-CM differentiation to model human cardiomyogenesis. First, the expression of *GATA4*, *PRDM1* and *TBX2* was examined during normal iPSC-CM differentiation. Consistent with previous studies, *GATA4* expression is observed from the onset of differentiation with a noticeable increase in expression at day 4 (Fig. 3A), coincident with the establishment of the early CP pool (Churko et al., 2018). Its expression rises throughout differentiation, peaking approximately when beating becomes apparent (days 9 and 10 of differentiation). Expression of *PRDM1* peaks early at day 3 and decreases when *GATA4* expression initially peaks at day 4 (Fig. 3A). Conversely, *TBX2* expression becomes prominent only after the peak in GATA4 expression. The expression patterns of these genes support the notion that the regulatory relationship between *GATA4*, *PRDM1*, and *TBX2* may be a conserved feature of cardiogenesis.

To establish whether the expression pattern of *PRDM1* and *TBX2* depends on GATA4, CRISPR-Cas9 gene editing was used to inactivate *GATA4* (Fig. S4). Knockout of the protein was confirmed by western blotting (Fig. 3B). RNA-seq and reverse transcription PCR (RT-PCR) were then used to examine the expression of the target genes in the GATA4-null line. As shown in Fig. 3C,D, the expression of *PRDM1* and *TBX2* is disrupted in the absence of GATA4, with *PRDM1* expression being higher than normal and *TBX2* expression being lower than normal. These results are consistent with the hypothesis that GATA4 regulates *TBX2* and *PRDM1* in cardiogenesis in humans as well as in *Xenopus*.

### GATA4-deficient iPSCs fail to form functional CMs

**Fig. 4.**
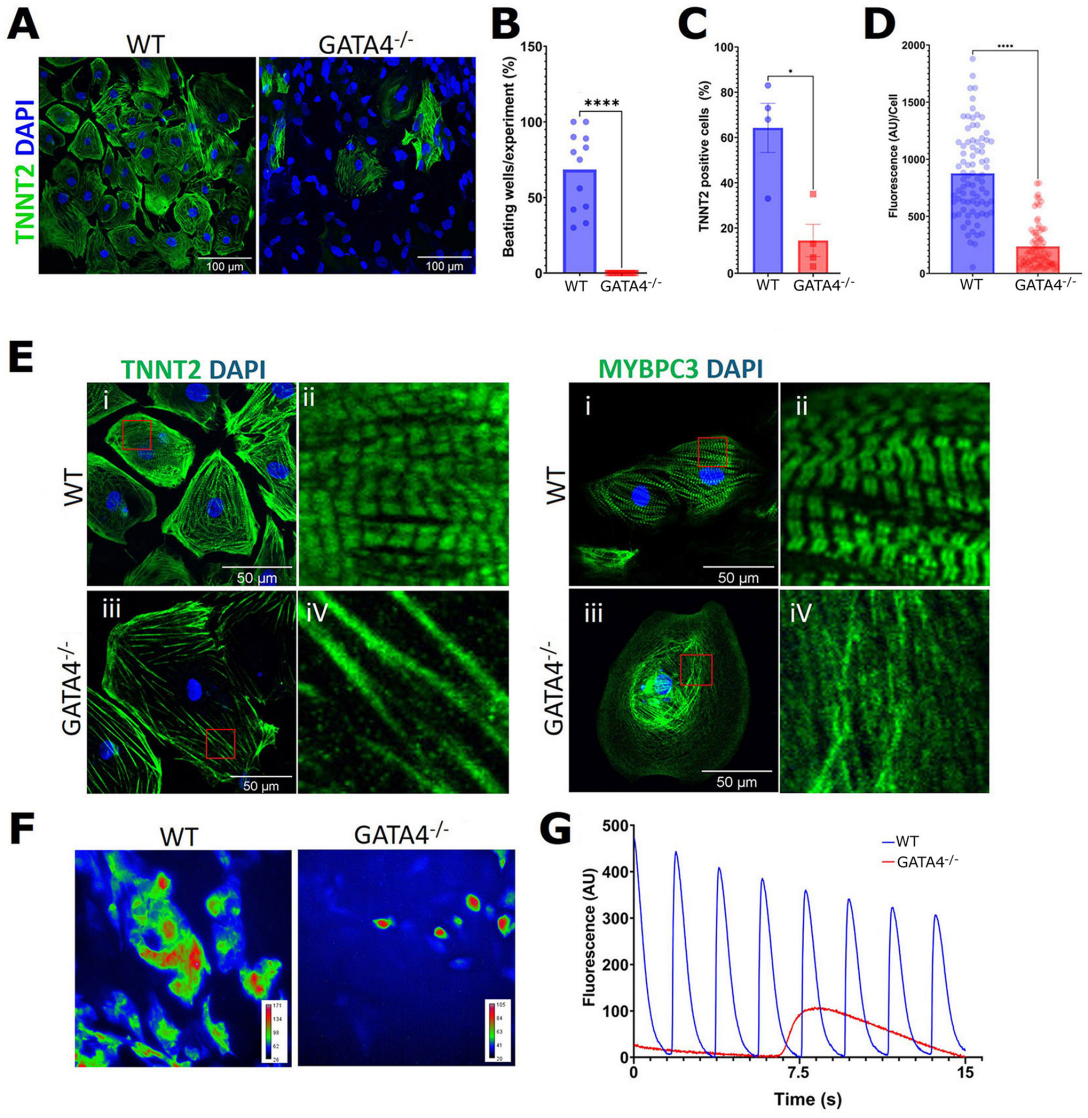
**GATA4-null iPSCs fail to form normal CMs.** (A) IF staining for TNNT2 (green) in WT and GATA4^−/−^ mutant cells at day 32 of cardiac-directed differentiation. DAPI (blue) was used to counterstain nuclei. Representative examples from four biological repeats are shown. The image for WT cells is shown at a higher magnification in E. (B) Proportion of beating wells per experiment in WT (153 wells) versus GATA4 null (139 wells) across 12 independent differentiation experiments. (C) A comparison of the proportion of TNNT2^+^ cells identified by IF between the cell lines. TNNT2^+^ WT cells=64.25±10.87% (*n*=935 cells) and GATA4^−/−^=14.50±7.14% (*n*=642 cells). Across four biological repeats. (D) TNNT2 mean fluorescence per cell for WT (*n*=81 cells) and GATA4^−/−^ (*n*=90 cells). Across four biological repeats. For panels B and C, a one-way ANOVA was used for statistical analysis. For panel D, a Student's *t*-test has been used. *P≤0.05 and ****P≤0.0001. (E) Representative images of individual WT and GATA4^−/−^ cells at 40× magnification stained for TNNT2 or MYBPC3 (green). DAPI (blue) was used to counterstain nuclei. The enlarged images outlined in red boxes provide a closer look at individual myofibrils. In the WT line, >90% of the CMs present have clear sarcomeres compared to <7% for the GATA4^−/−^ line. Further examples are presented in Fig. S6. (F) Calcium fluctuation was measured over time in a field of WT and GATA4^−/−^ cells at day 32 of differentiation using Fluo-8 AM. Signal variance over time is shown as an RGB pseudo-coloured projection, where red indicates the highest variance and blue the lowest. (G) In addition to these overviews, calcium flux was plotted over time from discrete regions of interest. For F and G, the images shown are representative of *n*=9 wells per cell line across three biological replicates. AU, arbitrary units.

The GATA4-null iPSC lines were differentiated using Wnt modulation protocols (Burridge et al., 2014; Lian et al., 2012) and were examined up to day 32 of differentiation. No beating was observed in 12 independent differentiation experiments. Staining for TNNT2 by immunofluorescence (IF) was used to detect CMs in iPSC-CM differentiation of wild-type (WT) and GATA4-null lines. On average, there was a fourfold to eightfold decrease in the number of TNNT2-positive cells (TNNT2^+^) formed from GATA4^−/−^ iPSCs in comparison to WT (Fig. 4C). In the few mutant cells that expressed TNNT2, the signal also appeared weaker. Mean fluorescence per cell was used to quantify this, and the average signal in the GATA4^−/−^ TNNT2^+^ cells was found to be 2.5 times lower than for WT TNNT2^+^ cells (Fig. 4D). Examination of the cells at a higher magnification reveals that in the TNNT2^+^ GATA4^−/−^ cells, myofibrils lack a normal striation pattern (Fig. 4E). Consistent with the results seen for TNNT2, staining for additional sarcomeric markers MYBPC3 (Fig. 4E) and filamentous actin (Fig. S6A) was also disrupted in the GATA4-null lines. In contrast, staining for general cytoskeletal protein α-tubulin (TUBA1A) appears normal (Fig. S6B).

The physiological activity of the WT and GATA4^−/−^ cells was examined at day 32 of differentiation using calcium imaging. Fig. 4F shows that calcium flux can be observed across a large proportion of WT cells; conversely, in the GATA4^−/−^ cells, this activity is restricted to a few small regions (Fig. 4F). When regions of interest for each are plotted individually, it is apparent that calcium cycling in the WT population is frequent and regular (Fig. 4G). In the GATA4-null cells, the transients detected were on average six times slower than their WT counterparts. This, together with the low differentiation efficiency, rare and low TNNT2 expression, and lack of sarcomere formation in the cells lacking GATA4, accounts for the failure to form beating CMs.

### GATA4-null cells likely adopt alternative cardiac cell fates

The vast reduction in the number of CMs (TNNT2^+^ and MYBPC3^+^ cells) in mutants raises the question of the remaining cell types' identity.

To address how GATA4-null lines diverge from the WT path of differentiation, RNA-seq was carried out on cells at days 2, 5, and 10 of differentiation. At day 2, the expression of GATA4 is very low in the WT line; therefore, a large effect on the transcriptome at this time was not expected. Indeed, the GATA4-null cell transcriptome appears to be comparable to that of the WT cells (Fig. S5A,B), as is the expression of early mesodermal and cardiac mesoderm markers *T* and *MESP1*, respectively (Fig. S5F).

**Fig. 5.**
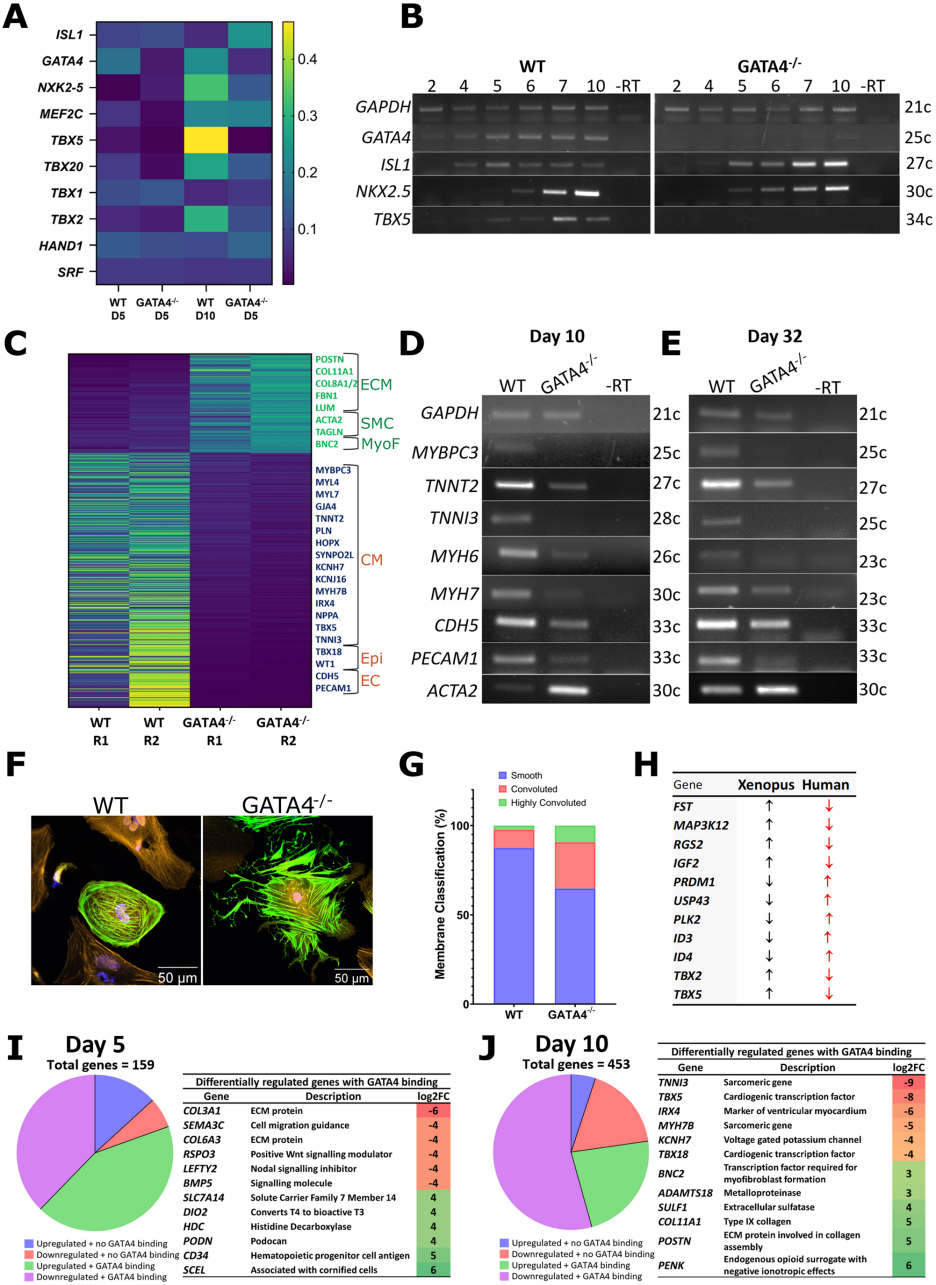
**In GATA4^−/−^ cells, CM and endothelial cell transcriptomes are suppressed, whereas smooth muscle cell and fibroblast genes are upregulated.** (A) A heatmap showing the normalised expression of a selection of marker genes indicative of progression through iPSC-CM differentiation. (B) Analysis of a number of CP marker genes by RT-PCR: expression of *GAPDH* was used for normalisation. (C) A heatmap showing the normalised expression of a selection of CM, myofibroblast (MyoF), endocardial (EC), ECM and smooth muscle cell (SMC) genes at day 10. (D,E) A selection of the genes from C was validated in an additional RNA set at days 10 (D) and 32 (E) of differentiation using RT-PCR; for A-E, the results generated are based on two biological repeats. (F) Immunofluorescent images showing WT and GATA4^−/−^ cells at 40× magnification at day 32 of differentiation. Stained for TNNT2 (green), cell mask orange (orange), and nuclei stained with DAPI (blue). (G) Cell membrane morphology was classified for WT and GATA4^−/−^ cells into smooth, convoluted, and highly convoluted. Those belonging to the convoluted category have membrane extensions, and those in the highly convoluted category display many of these branching extensions. WT cells analysed=88, GATA4^−/−^=55, over four biological repeats. (H) A table showing the direction of change for a selection of prospective GATA4 targets identified in the *Xenopus* screen (GATA4 overexpression) and their expression change in the GATA4-null iPSC line. Upregulated genes in the *Xenopus* column would be expected to be downregulated in human samples, and vice versa. (I-J) The pie charts displayed show the number of DEGs in the GATA4 mutants, at days 5 and 10, respectively, with a log2FC value of 2 or more that are bound by GATA4 in foetal or adult mouse hearts, according to datasets generated by Akerberg et al. (2019). The accompanying tables list a selection of these bound genes and their log2FC value from our RNA-seq analysis of the GATA4^−/−^ line.

At day 5 in the WT population, the expression of CP genes, including GATA4, rises. The levels of these genes are lower in the GATA4-null population, and at day 10, the discrepancy between the WT and GATA4-null lines becomes exaggerated. At day 10, the most affected cardiac GRN member is *TBX5*, being almost undetectable in the GATA4^−/−^ cells (Fig. 5A,B). However, not all members of the cardiac GRN are downregulated: for example, *MEF2C* expression is expressed at a similar level in both lines, and *ISL1* expression is increased, strongly suggesting that members of cardiac GRN display differential requirements for GATA4 function (Fig. 5A,B). Analysis performed at additional time points demonstrates that *TBX5* expression is undetectable during CM differentiation of the GATA4-null line (Fig. 5B). In contrast, the expression of *NKX2-5* is readily detectable, and the expression of *ISL1* is higher after day 6 (Fig. 5B).

During directed differentiation of iPSCs to CMs, other cardiovascular cell types are formed such as fibroblasts, endothelial cells, and smooth muscle cells (Grancharova et al., 2021; Jiang et al., 2022). Therefore, these cell types are the most likely candidates for the observed non-CMs in the GATA4-null mutant cell population.

In agreement with previous report that has identified an essential requirement for GATA4 in the formation of the epicardium (Watt et al., 2004), a decrease in the expression of epicardial marker genes (*TBX18* and *WT1*) was observed (Fig. 5C). Furthermore, the expression of endothelial marker genes *CDH5* and *PECAM1* was decreased to a similar degree as what was seen for the CM-specific gene set, suggesting that CMs, epicardial cells and endothelial cells all require GATA4 for their development in this model (Fig. 5C-E). The expression of markers that are highly but not exclusively expressed in smooth muscle cells such as *VIM*, *TAGLN*, and *ACTA2* was upregulated in the GATA4^−/−^ mutant cells (Fig. 5C-E). The marker of activated fibroblasts, *POSTN* (Fig. 5C), was also upregulated, as well as numerous extracellular matrix (ECM) components (Fig. 5C; Table S2). Our gene enrichment analysis indicates significant over-representation of multiple ECM terms, consistent with the upregulation of smooth muscle and fibroblast markers. Among the genes upregulated in GATA4^−/−^ cells at day 10 is *BNC2*, a recently described master regulator of myofibroblast differentiation (Fig. 5C) (Bobowski-Gerard et al., 2022). A closer inspection of cellular morphologies of mutant TNNT2^+^ cells revealed an appearance of highly convoluted cells suggestive of a myofibroblast phenotype (Fig. 5F).

Together, these results suggest that the loss of GATA4 adversely affects the development of CMs and endothelial lineages, whilst simultaneously promoting the development of related mesenchymal cells – activated fibroblasts, myofibroblasts and smooth muscle cells.

To see if GATA4 could directly regulate DEGs, we analysed the high-resolution GATA4 chromatin immunoprecipitation sequencing (ChIP-seq) dataset for adult and foetal mouse hearts (Akerberg et al., 2019). From our dataset at day 5, 159 DEGs were found to be consistently differentially regulated by a log2FC value of ≥2. Of these genes, 80% were associated with ≥1 GATA4-binding sites. At day 10, by these criteria, 453 genes were found to be differentially regulated, and of these, 77% were found to be associated with ≥1 GATA4-binding sites (Fig. 5I-J). Importantly, these included genes differentially expressed in both *Xenopus* and human RNA-seq datasets (Fig. 5H), suggesting that GATA4-regulated GRN has more extensive conservation besides *TBX2* and *PRDM1*. Both upregulated and downregulated DEGs were identified as direct targets based on GATA4 binding, in agreement with known activities of GATA4 in both transcriptional repression and activation.

### *TBX2* is required for normal CM differentiation

**Fig. 6.**
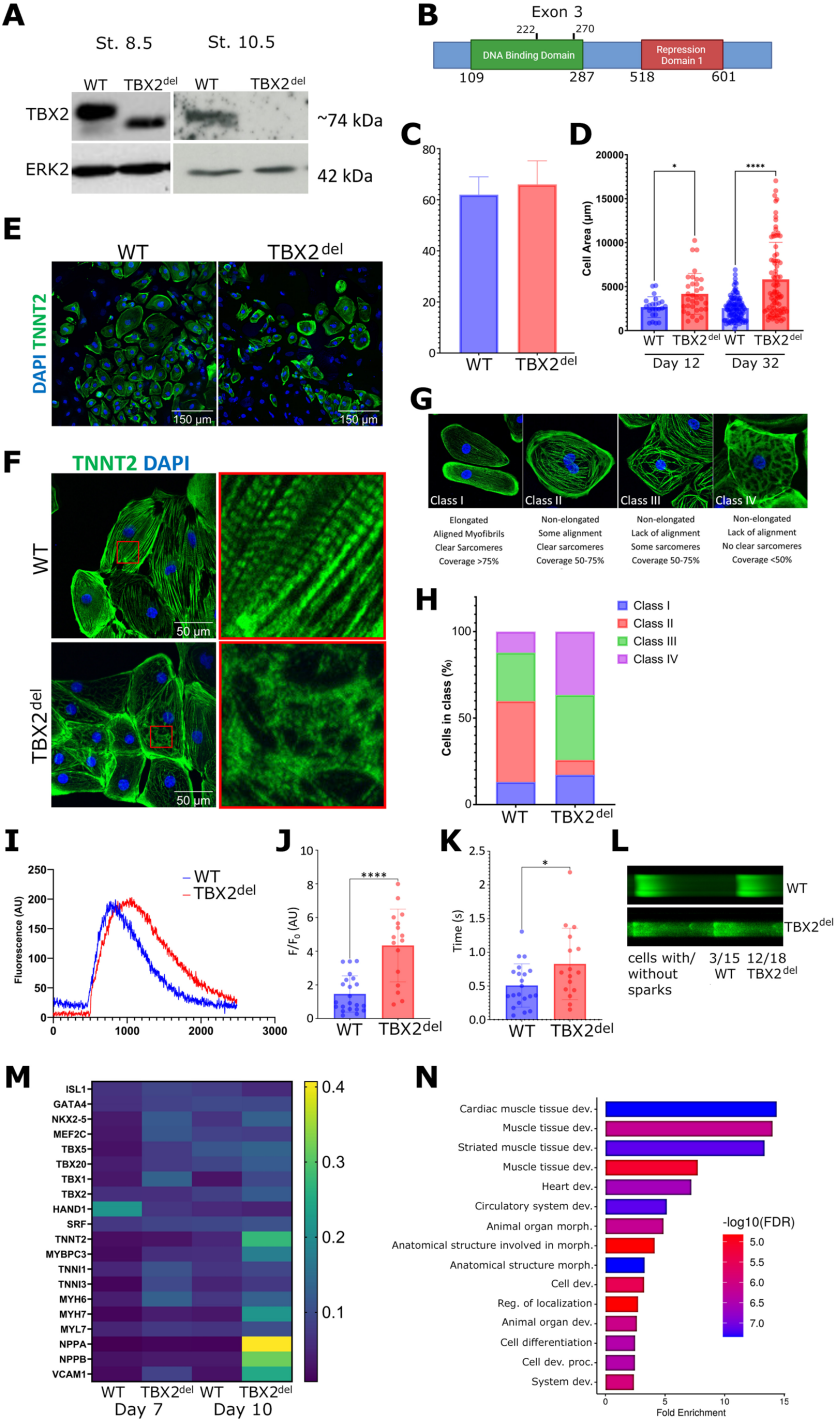
***TBX2* mutant cells display hypertrophic-like features consistent with maladaptive remodelling.** To better visualise annotations in the figure, the full name TBX2^c.700_761del^ is abbreviated as TBX2^del^. (A) A western blot showing TBX2 expression in *Xenopus* embryos injected uniformly with WT *TBX2* or *TBX2*^c.700_761del^ mutant mRNA at two- to four-cell stage. The embryos were harvested for protein analysis at stages 8.5 (blastula) and 10.5 (early gastrula). ERK2 was used as a loading control. The mutant protein is readily detected at st. 8.5 but is subsequently degraded at st. 10.5. (B) The TBX2 protein structure is shown with the DNA-binding domain indicated in green. In *TBX2*^c.700_761del^ mutants, exon 3 is skipped. The region of the TBX2 DNA-binding domain affected by this is indicated. Created in BioRender by Graham, N. (2026). https://BioRender.com/s50p7yi. This figure was sublicensed under CC-BY 4.0 terms. (C) The proportion of TNNT2^+^ cells in the total cell population was recorded. The values are given as a percentage±s.e.m. and are as follows: WT 62±7.00% (*n*=649 cells) and *TBX2*^c.700_761del^ 66±9.23% (*n*=871 cells), across *n*=3 biological replicates. (D) The surface area of the CMs was measured across three differentiations at day 32 and one differentiation at day 12. (E) Representative fields of WT and *TBX2* mutant day 32 iPSC-CMs are shown at 40× magnification. The cells were stained for TNNT2 (green) to identify CMs and counterstained with DAPI (blue) to identify nuclei. (F) Enlarged pictures of WT and *TBX2* mutant CMs at day 32 showing disarray of myofibrils and sarcomeres. (G) Representative images of cells belonging to classes I-IV, with class I cells showing the highest level of order and class IV the most severe disorganisation. Class II cell image is from Fig. 5F. (H) Quantification of CM disorganisation in the WT (*n*=107 cells) and *TBX2* mutant (*n*=93 cells) line through categorisation into one of the four categories described in G across *n*=3 biological repeats. (I) Representative Ca^++^ transients for WT and *TBX2*^c.700_761del^ mutants at ∼day 32 of differentiation for each cell line. (J) The amplitude of individual Ca^++^ cell transients. (K) Calcium imaging time to peak measurements. A one-way ANOVA has been used for all statistical comparisons shown. *P≤0.05 and ****P≤0.0001. (L) (upper panel). Exemplar confocal Ca^++^ line scan recordings of Ca^++^ transients in a WT cell. Spontaneous Ca^++^ sparks are visible during diastole. (lower panel) *TBX2* mutant cells exhibit more diastolic Ca^++^ sparks than WT cells, although this was not statistically significant (*P*=0.09; χ^2^ test). All calcium-handling data were collected across three biological repeats. (M) The heatmap displays the normalised RNA-seq data for a selection of CP and CM differentiation markers, *n*=2 biological repeats. The heatmaps with additional DEGs are shown in Fig. S9. (N) A selection of GO terms found to be upregulated in the *TBX2*^c.700_761del^ mutant at day 10 of differentiation. Graph produced using ShinyGo.

We next examined whether the GATA4 target genes identified and validated in the *Xenopus* screen are themselves required for normal iPSC-CM differentiation. CRISPR-Cas9 gene editing of the *TBX2* locus caused an in-frame deletion of the DNA-binding domain, likely resulting in a loss-of-function mutation (Fig. 6A,B; Fig. S8). The *TBX2* mutant iPSC line produces CMs at a similar rate to WT iPSCs (Fig. 6C). However, the cells produced are on average 1.6-fold larger than WT cells at day 12 of differentiation. This difference is accentuated at day 32, with the size of the mutant cells being around 2.3-fold larger (Fig. 6D). Additionally, the examination of the myofibril and sarcomeric structure of the *TBX2* mutant CMs by IF (Fig. 6F-H) reveals them to be significantly more disorganised (74%) than their WT counterparts (40%).

In the *TBX2* mutants, the amplitude of the calcium peaks recorded was notably higher, and there was a larger range of amplitudes observed, indicating that there is a wider heterogeneity in the Ca^++^ handling properties of the mutant CMs (Fig. 6J-K). *TBX2* mutants also displayed increased propensity for formation of spontaneous calcium sparks, indicating defective calcium handling (Fig. 6L). Taken together, these results indicate that interfering with the TBX2 function promotes formation of CMs with hypertrophic-like maladaptive properties.

To further explore the phenotype of the cells, RNA-seq was conducted on samples taken at days 7 and 10. These time points correspond with the peak of *TBX2* expression during iPSC-CM differentiation and the onset of beating, respectively. Notable findings include increased expression at day 10 of *NPPA* and *NPPB* (Fig. 6M), genes whose elevated expression is known to be associated with heart failure (Tan et al., 2002). TBX2 is known to repress *NPPA* in the AVC (Habets et al., 2002); thus, increased *NPPA* expression in *TBX2* mutant iPSC-CMs strongly suggests that the mutation leads to loss of function. *VCAM1* is another gene that has been associated with heart failure and immune cell infiltration of the myocardium (Troncoso et al., 2021; Wang et al., 2021) and was also upregulated at day 10 (Fig. 6M). The increased expression of several sarcomeric genes, e.g. *TNNT2*, *MYBPC3*, and *MYH7*, in *TBX2* mutant cells is consistent with the hypertrophic-like phenotype (Fig. 6M). In agreement with this finding, GO terms related to CM development such as Cardiac Muscle Cell Development were enriched (Fig. 6N; Table S2).

### *PRDM1*-deficient iPSC-CMs appear structurally unaltered but show premature beating

**Fig. 7.**
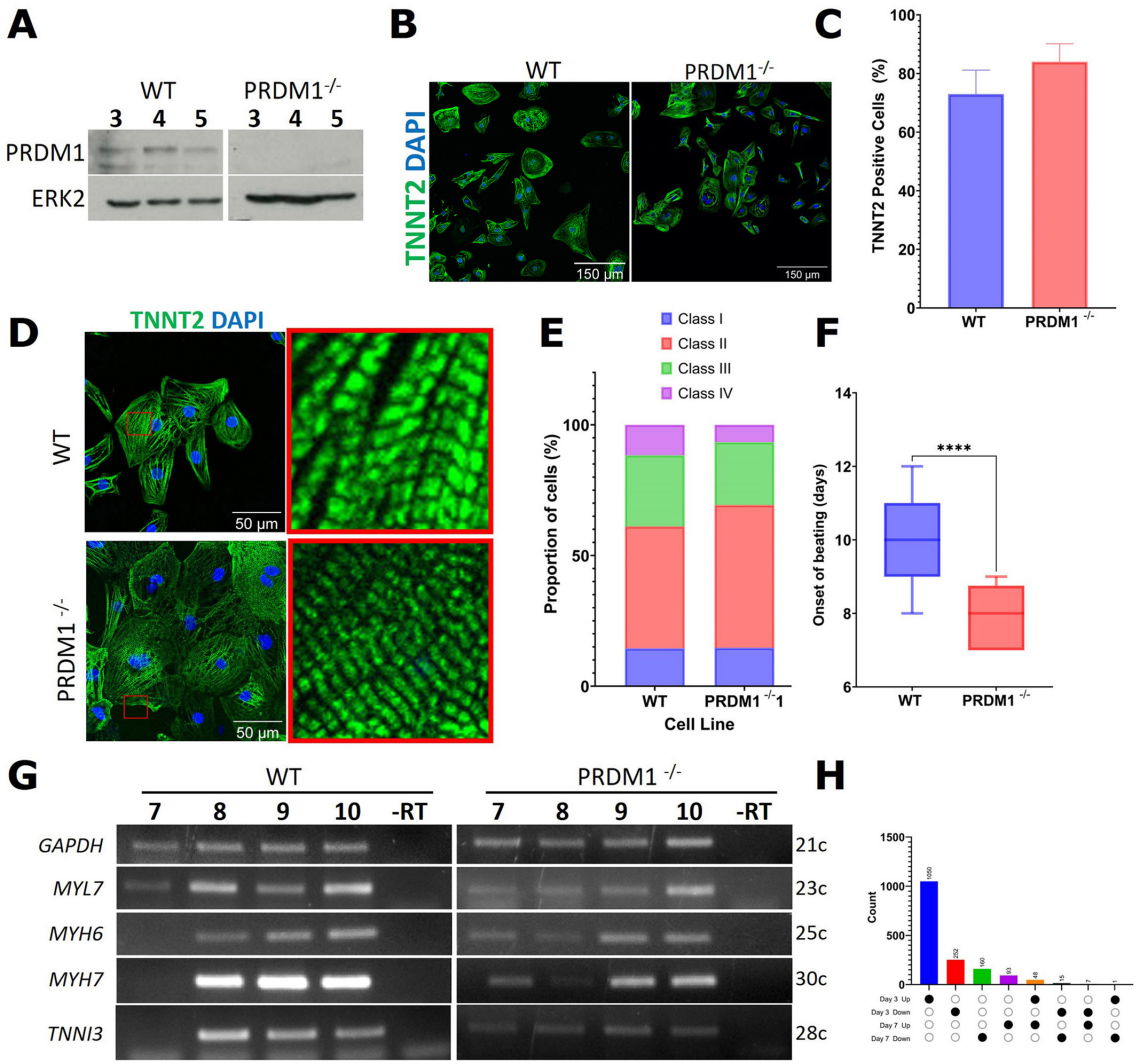
**Accelerated formation of CMs from *PRDM1***^−/−^ **iPSCs.** (A) Western blot analysis of PRDM1 protein in WT and mutant iPSCs at indicated days of iPSC-CM differentiation. ERK2 is used as a loading control. *n*=3 biological repeats. (B) Representative images of WT and *PRDM1*^−/−^ cells stained for TNNT2 (green) and counterstained with DAPI (blue) at day 32 of differentiation. (C) CM differentiation efficiency, assessed by staining for TNNT2 at day 10, is not affected in *PRDM1*^−/−^ cells. The values are given as a percentage±s.e.m. and are as follows: WT 73±16.3% *n*=576 cells; *PRDM1*^−/−^ 84±12.3% *n*=367 cells, across *n*=3 biological repeats. (D) Comparable sarcomeric structure was seen between the WT and PRDM1 mutant CMs. Staining for sarcomeric protein TNNT2 (green) in day 32 hCMs formed from WT and *PRDM1* mutant cells, counterstained with DAPI (blue); enlarged images are shown on the right-hand side. (E) The proportion of cells in each phenotypic class, WT *n*=180 and *PRDM1*^−/−^, *n*=150, over *n*=3 biological repeats. The classification system utilised is as described in Fig. 6. (F) Beating onset was recorded for WT (average onset=day 9.9, *n*=118 wells) and *PRDM1*^−/−^ (average onset=day 7.8, *n*=48 wells) lines across *n*=8 biological repeats. (G) Expression of a selection of sarcomere genes analysed by RT-PCR in a WT and *PRDM1*^−/−^ differentiation. *GAPDH* is used as a loading control. Days of iPSC-CM differentiation and the number of PCR cycles are indicated. (H) An UpSet plot showing the proportion of DEGs that are upregulated or downregulated at days 3 and 7 in the *PRDM1*^−/−^ line, as well as the overlap between these groups, *n*=1.

We next examined the requirement for *PRDM1* in iPSC-CM differentiation through the creation of a *PRDM1*-null line using CRISPR-Cas9 gene editing (Fig. S10). Knockout of the protein was confirmed by western blotting (Fig. 7A). The *PRDM1*^−/−^ line produced CMs at a similar efficiency to WT, and the phenotype of these cells when examined by IF resembles that of WT cells (Fig. 7C,D). In the *PRDM1*^−/−^ line, beating started around 2 days earlier on average than in the WT line (Fig. 7B). Gene expression analysis of several sarcomeric genes such as *MYL7*, *MYH6*, *MYH7*, and *TNNI3* indicates subtle mis-regulation, early increase at day 7 and subsequent decrease relative to the WT cells (Fig. 7E). Pilot RNA-seq results show that at day 3 when the level of *PRDM1* is highest, upregulated DEGs greatly outnumber downregulated DEGs (Fig. 7H) and include genes associated with neural development and pluripotency (Table S2), suggesting that the transcriptional repressor PRDM1 might be fine-tuning the timing and levels of gene expression during CM differentiation and repressing early alternative gene programmes.

## DISCUSSION

In this study, we used cardiogenic factors GATA4 and Nodal in pluripotent explants from *Xenopus* embryos to examine the cardiogenic gene programme. We identified a common gene set on which these cardiac-inducing factors converge to regulate cardiogenesis. We focused on TFs *prdm1* and *tbx2*, the expression of which in *Xenopus* embryos is regulated by GATA4, possibly directly. We find this regulatory relationship to be conserved in human cardiomyogenesis and that target genes *PRDM1* and *TBX2* can modulate the cardiac gene programme.

Our results demonstrate that GATA4 is essential for differentiation of human CMs from iPSCs. Previous work in mouse, *Xenopus* and zebrafish models has established distinct stage-dependent roles for GATA4 during CM development, with defective heart still being present in null mutant embryos (Borok et al., 2016; Crispino et al., 2001; Haworth et al., 2008; Holtzinger and Evans, 2005; Watt et al., 2004; Zeisberg et al., 2005). In the current study, we show that human *GATA4*^−/−^ iPSC lines have a greatly reduced capacity to form CMs and do not show beating activity, a phenotype that is stronger than the phenotypes gained from GATA4-null mutation in animal models. Furthermore, a recent report by Gonzalez-Teran et al. (2022) presented results for a human *GATA4*^−/−^ iPSC line that could form beating CMs, albeit at a greatly reduced frequency. The reasons for this apparent discrepancy are not clear but likely include experimental details such as genetic background, which is known to be a major determinant of phenotypic variation in human iPSCs (Kilpinen et al., 2017).

The failure of *GATA4*^−/−^ cells to form beating CMs in this study is underlined by a greatly reduced differentiation efficiency and defective sarcomere formation. Using genome-wide transcriptomic analysis, we documented the downregulation of CM and endothelial cell gene sets and an upregulation of genes associated with smooth muscle and activated fibroblast cell phenotypes. These include a marker of activated fibroblasts, *POSTN* (Shimazaki et al., 2008), and the recently described master regulator of myofibroblasts, *BNC2* (Bobowski-Gerard et al., 2022). A subpopulation of GATA4-null cells displayed a myofibroblast-like convoluted shape but was also found to be positive for TNNT2. The expression of CM-specific sarcomeric genes *TNNT2* and *MYBPC*3 has been identified in a subpopulation of human cardiac fibroblasts (Litviňuková et al., 2020). It will be of interest to investigate if loss of GATA4 promotes transition of those fibroblasts to a myofibroblast phenotype.

Loss of GATA4 also upregulates numerous genes involved in fibrosis and inflammation such as *COL3A1*, *COL8A1*, *COL11A1*, and *IL11* as well as *IGFBP7*, which is associated with senescence and heart failure (López et al., 2021; Moschen et al., 2011; O'Reilly, 2023; Xiang et al., 2017; Zhang et al., 2022).

The impact of GATA4 deficiency on cardiac GRN was not uniform: whilst the expression of *TBX5* was undetectable, the expression of *ISL1* (at day 10) and *NKX2-5* (at day 5) was upregulated in GATA4^−/−^ cells. This finding is consistent with the proposed repression of *NKX2-5* by GATA4/5/6 *in vivo* (Jiang et al., 1999). Of note, *NKX2-5* is known to be associated with ECM remodelling and a pathological smooth muscle cell phenotype (Papaioannou et al., 2024). It will be of interest to determine if *NKX2-5* has a similar role in regulating pro-fibrotic phenotype in GATA4^−/−^ iPSC-CM cells.

The downregulation of *TBX18* and *WT1* observed in GATA4^−/−^ iPSC-CM cells suggests defective epicardial development, in agreement with an *in vivo* requirement of GATA4 for development of the epicardium in the mouse embryo (Borok et al., 2016; Watt et al., 2004). This is an apparently paradoxical observation, as select markers of epicardial derivatives, smooth muscle and fibroblasts are upregulated in GATA4 mutant human iPSC-derived cells. One possibility is that diminished epicardial development still permits formation of abnormal smooth muscle cells and fibroblasts.

GATA4 is known to be expressed in cardiac fibroblasts. Our results show that deletion of GATA4 leads to a pro-fibrotic and myofibroblast gene expression signature, suggesting that it is required for the normal fibroblast phenotype. In agreement with this hypothesis, the overexpression of GATA4 in cardiac fibroblasts was recently shown to reduce heart-failure-induced fibrosis (Yamada et al., 2025). The role of GATA4 in attenuating fibrosis is not restricted to cardiac fibroblasts, as it also induces regression of liver fibrosis by deactivating hepatic stellate cells (Arroyo et al., 2021).

A potential mechanistic basis for the observed features of smooth muscle and activated fibroblasts in GATA4-null cells is offered by a recent study by Robbe et al. (2022), which demonstrated that GATA4 physically interacts with the repressive chromatin modifier CHD4, recruiting it to non-CM gene regulatory sites in mouse myocardium. The deletion of a GATA site in the modulatory region of smooth muscle gene *Myh11* led to its upregulation in CMs (Robbe et al., 2022). Misexpression of *Myh11* in CMs has previously been shown to result in sarcomere disarray, resulting in a significant decrease in ventricular output (Wilczewski et al., 2018). Thus, it is possible that removal of GATA4 leads to de-repression of non-CM genes which, based on our RNA-seq data, are part of gene expression signatures of smooth muscle cells and activated fibroblasts. Future work will determine the precise nature of non-CMs that express these genes.

Our results show that besides being a conserved target of GATA4 during cardiogenesis, *TBX2* is required for normal development of human iPSC-CMs. Mutant *TBX2* iPSC-CMs show hypertrophic-like phenotype at the cellular and molecular levels, including upregulation of CM stress genes as well as of CM differentiation gene sets. These features are consistent with the described role of TBX2 as a transcriptional repressor of the myocardial gene programme: in the absence of WT TBX2 activity, tight regulation of CM differentiation might be disrupted to result in upregulation of CM gene expression, leading to a hypertrophy-like phenotype.

The actions of *TBX2* in cardiac development have been well characterised in the context of AVC and outflow tract (OFT) formation, where it represses the myocardial gene programme (Aanhaanen et al., 2011; Christoffels et al., 2004; Harrelson et al., 2004; Singh et al., 2012). However, *TBX2* is broadly expressed in the linear heart tube before becoming restricted to these specialised tissues, indicating a potential for a broader role in early cardiac development outside of the OFT and AVC. In mouse *Tbx2* KO models, AVC and OFT development is defective, whilst there was no effect on the development of the myocardium (Harrelson et al., 2004; Singh et al., 2012). It is possible that the apparent discrepancy is caused by a species-specific difference or by the difference between *in vivo* and *in vitro* models. The Wnt modulation method of cardiac differentiation used in this study primarily produces ventricular-like CMs, with some atrial and node-like cells also present (Lian et al., 2012; Burridge et al., 2014). With differentiation protocols for the derivation of additional CM subtypes such as atrial, node-like, or AVC and conduction-like (Cyganek et al., 2018; Lyra-Leite et al., 2022; Prodan et al., 2022; Wiesinger et al., 2022; Ye et al., 2024), it will be possible to more precisely dissect any cardiac-cell-type-specific roles of human *TBX2*.

Loss of *PRDM1* expression did not have a major impact on cardiac differentiation in human iPSC-CMs but did subtly accelerate beating onset. In mutant cells, most DEGs appear to be upregulated at day 3 of differentiation. This is consistent with PRDM1 repressing differentiation in other developmental contexts, including mammary luminal and sebaceous gland stem cells; in migrating primordial germ cells; and in enterocyte maturation (Ahmed et al., 2016; Harper et al., 2011; Horsley et al., 2006; Yamashiro et al., 2016). Therefore, in this scenario, it is possible that the absence of PRDM1 leads to precocious CM beating through a lack of repressive activity, which might be required for tightly regulated progression through iPSC-CM differentiation.

### Limitations

A clear limitation of the current study is the lack of high-resolution single-cell RNA-seq (scRNA-seq) data on cell fate trajectories in GATA4^−/−^ iPSC-CM cultures, which could provide better insight into the roles of GATA4 in specification and differentiation of cardiovascular cell types.

## MATERIALS AND METHODS

### *Xenopus* embryos and explants

All work with *Xenopus laevis* was approved by Cardiff University's Animal Welfare and Ethical Review Board and was undertaken under a license from the UK Home Office. *X. laevis* embryos were obtained by mating of frogs primed with human chorionic gonadotrophin (Sigma) or by *in vitro* fertilisation (Sive, 2000). Jelly membrane was removed with 2% cysteine-HCl, pH 7.8 (Sive, 2000). Embryos were grown in 10% normal amphibian media (NAM) and staged as described (Sive, 2000). Whole embryos or explants were cultured until age-matched control siblings had reached the desired stage. Microinjections were carried out using an IM-300 Microinjector (Narishige Scientific) in 75% NAM containing 3% Ficoll (Sigma). MOs were supplied by Gene Tools (http://www.gene-tools.com/) and injected at 10 nl/embryo. Tbx2 MO was described (Cho et al., 2011). GATA4 and GATA5 translation and splicing blocking MOs were described (Haworth et al., 2008). A mMESSAGE mMACHINE kit (Ambion) was used for capped mRNA synthesis. Previously described templates for making capped mRNA are as follows: GATA4-GR (Afouda et al., 2005; Latinkić et al., 2003), *Gata4* (Gallagher et al., 2012), and *Xenopus nodal5* (Takahashi et al., 2000). *Xenopus prdm1* (de Souza et al., 1999) was obtained from the European Xenopus Resource Centre (Portsmouth, UK), and the coding sequence was amplified by PCR and cloned into *Bam*HI-*Xba*I sites of pCS2+ by PCR. WT and mutant human *TBX2* CDS were cloned into pCS2+ using PCR. The correct sequence was confirmed by sequencing. Injection solutions included lineage tracers biotin- and rhodamine-dextran (Invitrogen; Latinkić et al., 2003).

For cardiogenic Nodal signalling, we injected 50-110 pg of *nodal5* mRNA, and for lower, non-cardiogenic Nodal signalling, we used 16 units of activin protein provided as XTC-cell-line-conditioned media (Smith et al., 1988). Cardiogenic *nodal5* and non-cardiogenic but muscle-inducing activin doses were confirmed (Fig. S1A). For the RNA-seq experiment, ∼50 explants per sample were used, and for validation experiments, 20-30 explants per sample were used.

### Chromatin immunoprecipitation

Chromatin immunoprecipitation was performed as described by Blythe et al. (2009) with minor changes: two to 300 embryos were injected at the two-cell stage with 0.4 ng of capped mRNA coding for HA-tagged rat GATA4 (Gallagher et al., 2012). Stage 9-9.5 embryos were fixed in a 50 ml Falcon tube for 30 min in 25 ml of 10% NAM and 1% formaldehyde on ice without agitation, briefly washed in 10% NAM and quenched for 10 min in 25 ml of 10% NAM and 0.125 M glycine, followed by three washes in 10% NAM. Embryos were transferred into 1.5 ml microcentrifuge tubes (50/tube) and frozen on dry ice before storage at −80°C.

The crosslinked 50 embryos were thawed on ice for 15 min and homogenised in 600 ml of RIPA buffer+protease inhibitor (PI) tablets (complete EDTA-free, Roche) and incubated for 15 min, beginning from the time of initial homogenisation. Homogenates were centrifuged for 5 min at 730 ***g*** and 4°C. The supernatant was removed, and the inside wall of the tube above the pellet was wiped with fine paper to remove the lipid residue. The pellet was resuspended in 400 ml of RIPA buffer+PI to obtain ∼450 ml. Sonication was performed with Soniprep 150 at 70% power: eight cycles (10 s on and 50 s off) in 2 ml round-bottom Eppendorf tubes on an ice water bath. The shear median DNA size is ∼500 bp. The volume was adjusted to 650 ml with RIPA+PI, and samples were centrifuged for 10 min at 13,000 ***g*** and 4°C. For the supernatant, 600 ml was taken for IP, 5 ml for the input, and 20 ml for the shear control. Anti-HA antibody magnetic beads were used according to the manufacturer's recommendations (Pierce). Fifty microlitres of beads per 50 embryos was prewashed three times for 5 min in TBS+0.01% BSA using a magnet stand. A 600 ml aliquot of the supernatant was taken for IP, mixed with prewashed beads and incubated overnight with rotation at 4°C. Supernatants were removed, and beads were stored at −20°C. Beads were washed three times for 5 min in TBS-Tw (0.01% Tw20). For reversing crosslinks, beads (with chromatin), input and shear control samples were supplemented with 200 ml of TE buffer+1/20 vol. of 5 M NaCl and incubated overnight at 65°C. An aliquot of 1 ml RNase A (10 mg/ml) was added to each sample, which was then incubated for 30 min at 37°C. Subsequently, 1 ml of proteinase K (Roche) was added, and the sample was incubated for 1 h at 55°C. Proteins were removed by phenol-chloroform extraction, and DNA was precipitated with isopropanol. Samples were dissolved in 50 ml of ultrapure distilled water. PCRs were performed as for RT-PCR (below). Amplicons were chosen within 10 kb upstream and 2 kb downstream of the translation start site based on the presence of at least one consensus GATA-binding site.

### RNA extraction and RT-PCR analysis

RNA was extracted using the acid-guanidinium thiocyanate phenol chloroform method (Chomczynski and Sacchi, 1987), and cDNA was synthesised using the RevertAid reverse transcriptase kit (Thermo Fisher). All primer sequences used were designed using NCBI's Primer-BLAST program and are listed in Table S4. Samples were normalised using *odc1* primers for *Xenopus* samples and *GAPDH* primers for human samples. To determine the appropriate amount of cDNA for subsequent PCRs, reactions were conducted in a 25  µl volume containing 0.1 µM each of forward and reverse primers (Eurofins), 1× MyTaq Red reaction buffer (Bioline), 0.5 U KAPA Taq Polymerase (KAPA Biosystems), and the required volume of cDNA. An MJ Mini Thermal Cycler Machine (Bio-Rad) was used for cycling. The amplicons generated were visualised using agarose gel electrophoresis. All primer sequences are presented in Table S4.

Whole-mount *in situ* hybridisation to detect the expression of *myl7* in *Xenopus* embryos was performed as described (Gallagher et al., 2012).

### Human iPSC culture and gene editing

All cell lines produced in this study were derived from the Rebl Pat iPSC line kindly provided by Professor Chris Denning, Nottingham University (Hammad et al., 2016).

### iPSC culture

iPSCs were cultured on Corning™ cell culture plates coated with Geltrex™ (Thermo Fisher) at 30-45 µg/ml in DMEM/F-12 media (Thermo Fisher) and maintained in B8 media formulated as described by Kuo et al. (2020). Passaging was carried out when cultures reached 75-80% confluency. ReLeSR™ was used to dissociate iPSC colonies as per the manufacturer's instructions (STEMCELL Technologies); following this, the cells were replated and fed with B8 2.5 µM Y27632 (Hello Bio) for 24 h following passaging before being switched to B8 for regular feeding every other day. All cells were maintained at 37°C in a humidified atmosphere with 5% CO_2_.

### CRISPR-Cas9 gene editing of iPSCs

All CRISPR RNAs (crRNAs) were designed as pairs using CCTop (Stemmer et al., 2015). Each pair was intended to be used together to induce a frameshifting mutation. To produce guide RNAs (gRNAs), each crRNA (Integrated DNA Technologies) was combined with a transactivating crRNA (tracrRNA) (Integrated DNA Technologies) at an equimolar concentration in IDTE nuclease-free buffer {30 mM [4-(2-hydroxyethyl)-1-piperazineethanesulphonic acid (HEPES)] (pH 7.5), 100 mM potassium acetate} and then heated to 95°C for 2 min followed by gradual cooling to room temperature (RT). Each gRNA was then combined with an equal volume of 6.2 µg/µl Alt-R S.p. HiFi Cas9 Nuclease V3 diluted in Cas9 storage buffer [10 mM Tris-HCl (pH 7.4), 300 mM NaCl, 0.1 mM EDTA, 1 mM dithiothreitol (DTT)] and incubated together for 10 min at RT to form a ribonucleoprotein (RNP) complex. Transfection of the RNPs into iPSCs was carried out in suspension using the P3 Primary Cell 4D-Nucleofector™ X Kit L (Lonza) and a Lonza 4D Nucleofector with program CA137. Single clones were selected and screened using gDNA PCR followed by gel electrophoresis. All mutations were confirmed using Sanger sequencing (Eurofins) and Synthego ICE (Conant et al., 2022) signal deconvolution. Sequences of crRNAs and genotyping PCR primers are provided in Table S4. Following gene editing, the pluripotency of the lines was assessed by confirming the expression of *NANOG*, *POU5F1* and *SOX2* (Figs S4, S5, S7 and S10).

### Karyotyping

Genomic DNA from the parental Rebl Pat human iPSC line and gene-edited lines derived from it was extracted from >2 million cells using the Blood and Tissue DNA Extraction Kit (Qiagen), following the manufacturer's guidance. The gDNA was karyotyped using the Infinium Global Screening Array-24, covering 654,027 markers across the genome at the MRC Centre for Neuropsychiatric Genetics and Genomics, Cardiff University. The results are summarised in Table S5.

### CM differentiation and maintenance

Differentiation of iPSCs into CMs was carried out using two commonly utilised Wnt modulation protocols (Burridge et al., 2014; Lian et al., 2012). For differentiation, cells were seeded at a density of 14×10^4^-17.5×10^4^ cells per cm^2^ in wells coated with Geltrex™ as above and expanded to 75-80% confluency in B8 medium before differentiation was initiated. For the CDM3 protocol published by Burridge et al. (2014), the cells were changed to CDM3 (RPMI 1640 500 µg/ml, L-ascorbic acid-2-phosphate 213 µg/ml, penicillin-streptomycin 50 U/ml) with 6 µM CHIR99021 (Hello Bio) for 48 h. Following this, the medium was changed to CDM3 with Wnt 2 µM WntC59 (Hello Bio) for 48 h. After these treatment stages, cells were maintained in CDM3 media and refreshed every other day and then switched to CDM3-L (RPMI 1640 no glucose, 500 µg/ml Fraction V BSA, 213 µg/ml L-ascorbic acid-2-phosphate, 5 mM sodium DL-lactate) from days 10 to 14 to metabolically enrich the cultures for CMs.

For the GiWi protocol by Lian et al. (2012), the cells were plated as above and changed to RPMI B27^-insulin^ (Thermo Fisher) with 6 µM CHIR99021 for 24 h to initiate differentiation. This medium was then removed and replaced with RPMI B27^-insulin^ for 48 h. Following this, the spent medium was removed and mixed at 1:1 ratio with fresh RPMI B27^-insulin^ to make ‘conditioned medium’. To the conditioned medium was added 2 µM WntC59, and the cells were maintained in this for a further 48 h. After this, the medium was refreshed every 2 days with RPMI B27^-insulin^ and then with RPMI B27 from days 8 to 10. For cells differentiated using the GiWi protocol, RPMI B27-L (RPMI 1640 no glucose, 1× B27, 5 mM sodium D-L-lactate) was used to metabolically select cells from days 10 to 14.

Upon finishing selection, CMs derived using the CDM3 or GiWi protocols were maintained in CDM3 with media changes every 2 days.

### CM dissociation

The cell layer was washed with Hanks’ Buffered Saline Solution without Ca and Mg (HBSS, Merck). This was then removed, a pre-warmed dissociation solution (HBSS, 200 U/ml Collagen Type II, Worthington Biotech; 1 mM HEPES, pH 7.4, 10 µM Y37632, Hello Bio) was added, and the cells were incubated at 37°C for 3-3.5 h. The cell solution was then gently triturated, combined with an equal volume of media, and then filtered through a 100 µm filter. The cells in suspension were spun down at 400 ***g*** for 5 min, and the supernatant was removed. The cell pellets were resuspended at an appropriate concentration in CDM3 with 10% heat-inactivated FBS for replating. The following day, the medium was changed to CDM3.

### Immunofluorescence

Any cells to be analysed were dissociated as described above. For each sample, three wells per plate were pooled to minimise well-to-well variation and then plated onto 13 mm glass coverslips coated with 30-45 µg/ml of Geltrex™. The cells were allowed to attach and recover for a minimum of 48 h before any further processing. The fixation and permeabilisation method used was dependent on the antigen detected (see Table S3): either being carried out using 4% PFA for 10 min at RT, followed by permeabilisation with 0.25% Triton-X-100 DPBS without Ca^++^ and Mg^++^ (Thermo Fisher) for 10 min at RT, or using 100% ice-cold methanol for 15 min at 4°C. Regardless of the fixation and permeabilisation method used, blocking was carried out with DPBS without Ca^++^ and Mg^++^ 10% serum for 1 h at RT, with serum type matched to the secondary antibody used. Primary antibody incubation was carried out overnight at 4°C in blocking buffer. The samples were then washed five times for 15 min at RT with PBS-0.1% Triton-X-100 and then incubated with 1:1000 goat anti-mouse or anti-rabbit Alexa Fluor-conjugated antibodies (Thermo Fisher) in blocking buffer overnight. Information on antibodies used is shown in Table S4.

Hoechst 33342 at 1 µg/ml was used to counterstain nuclei by incubation for 15 min, followed by three 5-min washes in PBS without Ca^++^ and Mg^++^. This was followed by mounting in VECTASHIELD hard set mounting media with Phalloidin-TRITC. Alternatively, HCS Cell Mask Orange (Invitrogen) was used at 1× to stain cell membranes and nuclei for 30 min, followed by three 5-min washes PBS without Ca^++^ and Mg^++^. Mounting was then carried out using VECTASHIELD with DAPI (Vector Laboratories) to stain nuclei. A Zeiss LSM880 confocal microscope was used for imaging and FIJI/ImageJ software (Schneider et al., 2012) for image analysis and editing. Following IF staining and imaging, the TNNT2^+^ cells were analysed against the criteria adapted from Ang et al. (2016).

### Calcium imaging

Any cells to be analysed were dissociated as described above and then plated onto 13 mm glass coverslips coated with 30-45 µg/ml of Geltrex™. The cells were allowed to attach and recover for a minimum of 48 h before any further processing. For analysis of the TBX2 lines and their WT counterparts, cells were loaded with the Ca^++^-sensitive indicator Fluo-4-AM (5 µmol/L) for 30 min at RT, followed by at least 10 min for de-esterification. Experiments were performed in a modified Tyrode's solution containing 133 mM NaCl, 5 mM KCl, 1 mM NaH_2_PO_4_, 10 mM HEPES, 10 mM glucose, 1.8 mM CaCl_2_, and 1 mM MgCl_2_. The solution was adjusted to pH 7.4 with NaOH. Confocal line-scanning microscopy was performed using an inverted Leica SP5 confocal microscope with a 63×1.2 NA water immersion objective. Spontaneous Ca^++^ transients were recorded using the 488 nm line of an argon laser in line-scanning mode at 400 lines per second, with fluorescence emission collected between 500 and 650 nm. The confocal pinhole was set to <2 Airy units. For the GATA4 lines and WT controls, imaging was conducted using the widefield setting on an Olympus IX71 microscope. Before imaging, the cells were loaded with 1 µM Fluo-8-AM or 1 µM Fluo-4-AM (Stratech) in cell culture medium for 30 min at 37°C. This medium was then removed, and Tyrode's solution with 1 mM HEPES pH 7.4 (Merck, T2397) was added. The cells were then imaged using a 488 nm laser. For both, the temperature was maintained at 37°C during Ca^++^ imaging experiments using a heated microscope enclosure. The change in fluorescence over time was plotted using FIJI/ImageJ software (Schneider et al., 2012), and Ca^++^ transient analysis was performed using custom-written MATLAB scripts. As part of this analysis, non-cell background fluorescence was subtracted from any readings, and then average cell fluorescence (F) was divided by the diastolic fluorescence (F_0_) to account for possible variability in dye loading. This was used to calculate an average for the Ca^++^ transient amplitude value (reported as F/F_0_) and full duration at half maximum (FDHM).

### RNA-seq

The *Xenopus* RNA samples were converted to cDNA libraries using a Bioo NEXTflex directional v.2 kit and were sequenced at Bristol University's sequencing facility. The human RNA samples were converted to cDNA libraries using the Illumina TruSeq stranded mRNA library kit and sequenced at the Genome Research Hub, School of Biosciences, Cardiff University.

For the iPSC-CM experiments, RNA samples were converted to cDNA libraries (using the Illumina Trueseq kit) and sequenced at the Genome Research hub, School of Biosciences, Cardiff University. *n* refers to RNA samples derived from independent experiments. For each sample, three wells per plate were pooled to minimise well-to-well variation. In the GATA4-null dataset, day 2 samples have *n*=1, whilst all other time points have an *n* of 2. All RNA-seq samples relating to the *TBX2* lines have an *n* of 2, and all *PRDM1* time points have an *n* of 1.

### RNA-seq quantification and differential expression

For *X. laevis* sequence analysis, paired-end reads were aligned to *X. laevis* gene models v7.2 by JGI with Bowtie (Langmead et al., 2009), allowing for up to three mismatches in seed and leaving the other parameters at the default value. Gene hits were counted using RSEM (Li and Dewey, 2011) with default parameters. Expected read counts reported by RSEM were used to call DEGs, which were called using DESeq (Anders and Huber, 2010). The dispersion was estimated with ‘per-condition’ method and ‘gene-est-only’ sharing mode. Each treatment was separately compared to control at each time point, and genes with adjusted *P*-value (padj) <0.1 were defined as differentially expressed. To determine a gene's differential expression irrespective of time point, we took the time point with the minimum padj.

For human sequence analysis, RNA-seq data (75 bp single end) generated from human iPSC cell lines (40-45 M per sample) were quality trimmed using fastp (Chen, 2023), and trimmed sequences were aligned against the annotated Ensembl GRCh38.p14 human genome using STAR (Dobin et al., 2013). Quantification against gene objects was performed using Rsubread (Liao et al., 2019), and DEGs were derived using DESeq2 through the SARTools wrapper with default parameters (Varet et al., 2016). As with *Xenopus* data, each time point was compared to time- and batch-matched controls, and genes with padj<0.1 were defined as differentially expressed.

### Gene set enrichment

For *X. laevis*, to assess the composition of each group of genes, we performed gene set enrichment using Enrichr. We took genes from each cluster with a known *X. laevis* gene symbol and converted these to human symbols by removing any ‘.N’, ‘-a’, or ‘-b’ suffix for an integer *N* and converting it to uppercase. We then made the following substitutions to convert certain known *Xenopus* gene symbols to human, where the name of the orthologue has diverged or only a paralogue exists: *pou5f3*→*POU5F1*, *mix1*→*mixl1*, *dppa2*→*DPPA4*, *lefty*→*lefty2*, *ventx1-3*→*NANOG*, *mespb*→*MESP1*, *sox17a/b*→*SOX17*. We remove any duplicate names that arose in this process. Gene nomenclature for *X. laevis* follows the guidelines described at Xenbase (xenbase.org) (Fisher et al., 2023). For simplicity, in figures, we omitted the information on homologues (.L or .S) with the exception of *lefty1.S*, which was validated as DEG, unlike *lefty1.L*. We calculated enrichments for the following gene sets: KEGG_2021_Human, WikiPathway_2023_Human, BioPlanet_2019, GO_Biological_Process_2023, GO_Molecular_Function_2023, GO_Cellular_Component_2023. All enrichments are found in Table S1.

Human GO enrichment analysis was carried out using ShinyGO v0.77 and STRING v11.5 for network analysis (Ge et al., 2020; Szklarczyk et al., 2019) and is presented in Table S2. Normalised counts for DEGs identified in human iPSC samples are presented in Table S3.

### Western blotting

Cells were lysed in RIPA buffer (50 mM Tris-HCl, pH 8, 150 mM NaCl, 0.1% SDS, 0.5% Na deoxycholate, 1% NP-40, and 1× PI Complete Mini, Roche). Total protein extracts were mixed with 2× Laemmli loading buffer (Bio-Rad) and boiled for 10 min before loading; 20 µg of total protein was used per well. Bands were resolved via polyacrylamide gel electrophoresis; the samples were then transferred to a polyvinylidene difluoride membrane (Millipore). To block the membranes for 1 h, 5% skimmed milk in TBS Tween 20 (TBS-Tw: 5 mM Tris-Base, pH 7.4, 20 mM NaCl, 0.1% Tween 20) was used. All antibodies used are described in Table S4. For detection of TBX2, skimmed milk concentration was reduced to 1% to improve signal intensity. Incubation with primary antibodies was carried out overnight in blocking buffer at 4°C with constant rotation. Following this, the membranes were washed with TBS-Tw three times for 10 min each. Secondary antibodies were then applied to the membranes diluted in 5% skimmed milk TBS-Tw or for detection of TBX2 1% skimmed milk TBS-Tw. The membranes were washed with TBS-Tw three times for 10 min each to remove excess antibody. Clarity Western ECL (Bio-Rad) was used for development, followed by exposure of the membranes to Amersham Hyperfilm (Merck).

## Supplementary Material



10.1242/biolopen.062553_sup1 Supplementary information

Table S1. Gene set enrichment analysis for the overlap in differentially expressed genes in *Xenopus* explants treated with Gata4 (G4), Gata4 and Dkk1 (G4D), or Nodal (N).

Table S2. Gene set enrichment analysis for the human iPSC lines at the days indicated.

Table S3. Normalised count from RNA-seq analysis of the WT Rebl Pat parental human iPS cell line and its mutant derivatives at different days of iPSC-CM differentiation.

Table S4. Information on antibodies, sgRNAs and primers used in this study.

Table S5. Karyotyping data for the parental Rebl Pat iPS cell line and its edited derivatives
