# Supplementary material for: Conserved roles of GATA4 and its target gene TBX2 in regulation of human cardiogenesis
Source: Biol Open. 2026 Jun 15;15(6):bio062553. doi: 10.1242/bio.062553 (PMC13312917; doi:10.1242/bio.062553)
Supplement: Supplementary information [file biolopen-15-062553-s1.pdf]

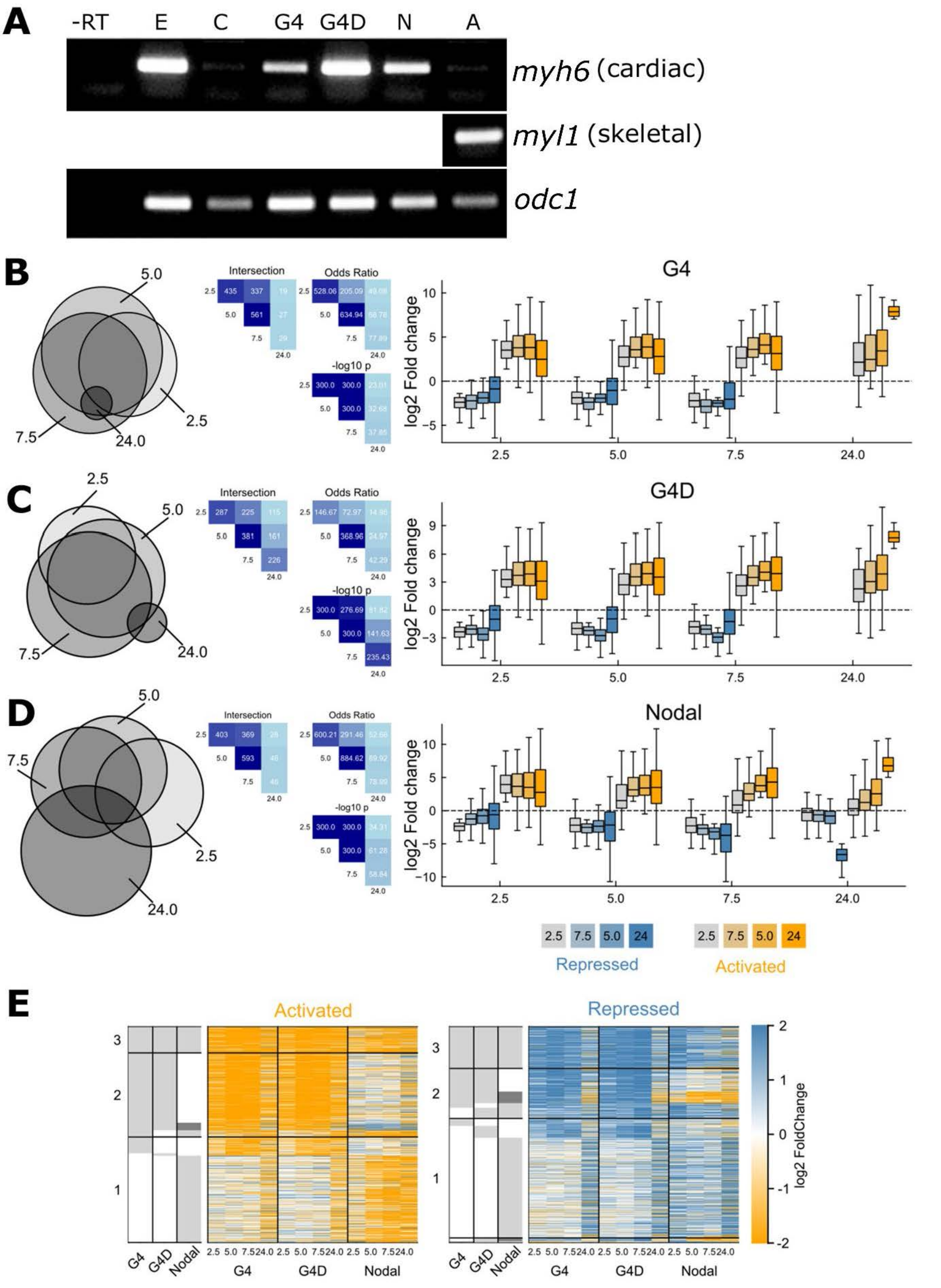

Fig. S1. Summary of differential expression time course for genes induced by GATA4 (G4), GATA4+DKK1 (G4D), and Nodal5 (N, Nodal) in *Xenopus* animal cap explants. (A) Confirmation of cardiogenic conditions used in RNA-seq. RT-PCR showing expression of

Biology Open • Supplementary information

cardiac marker *myh6* in animal cap explants exposed to cardiac inducing conditions (G4, G4D and Nodal) and *myl1* when skeletal muscle is induced by a low dose of Activin protein. *odc1* is used as a loading control. Samples were collected when sibling embryos reached st. 34. E- sibling embryo. C- control animal caps. (B-D). Intersection between differentially expressed genes between timepoints for G4 (B), G4D (C), Nodal (D). For each condition, left provides Venn diagram of intersection between differentially expressed genes at each timepoint; center provides intersection size, odds ratio, and right-tail Fisher exact test p-values between intersection of all pairs timepoints; right boxplots describing the log2 Fold Change of genes differentially expressed at each time point (horizontal axis 2.5, 5.0, 7.5, and 24h), at all other timepoints. We find highly significant associations between all timepoints, particular 2.5-7.5h. (E) Heatmap of log fold change of activated and repressed genes per timepoint, as in Figure 1D regardless of timepoint. Left grey heatmap indicates whether gene is differentially expressed at any timepoint.

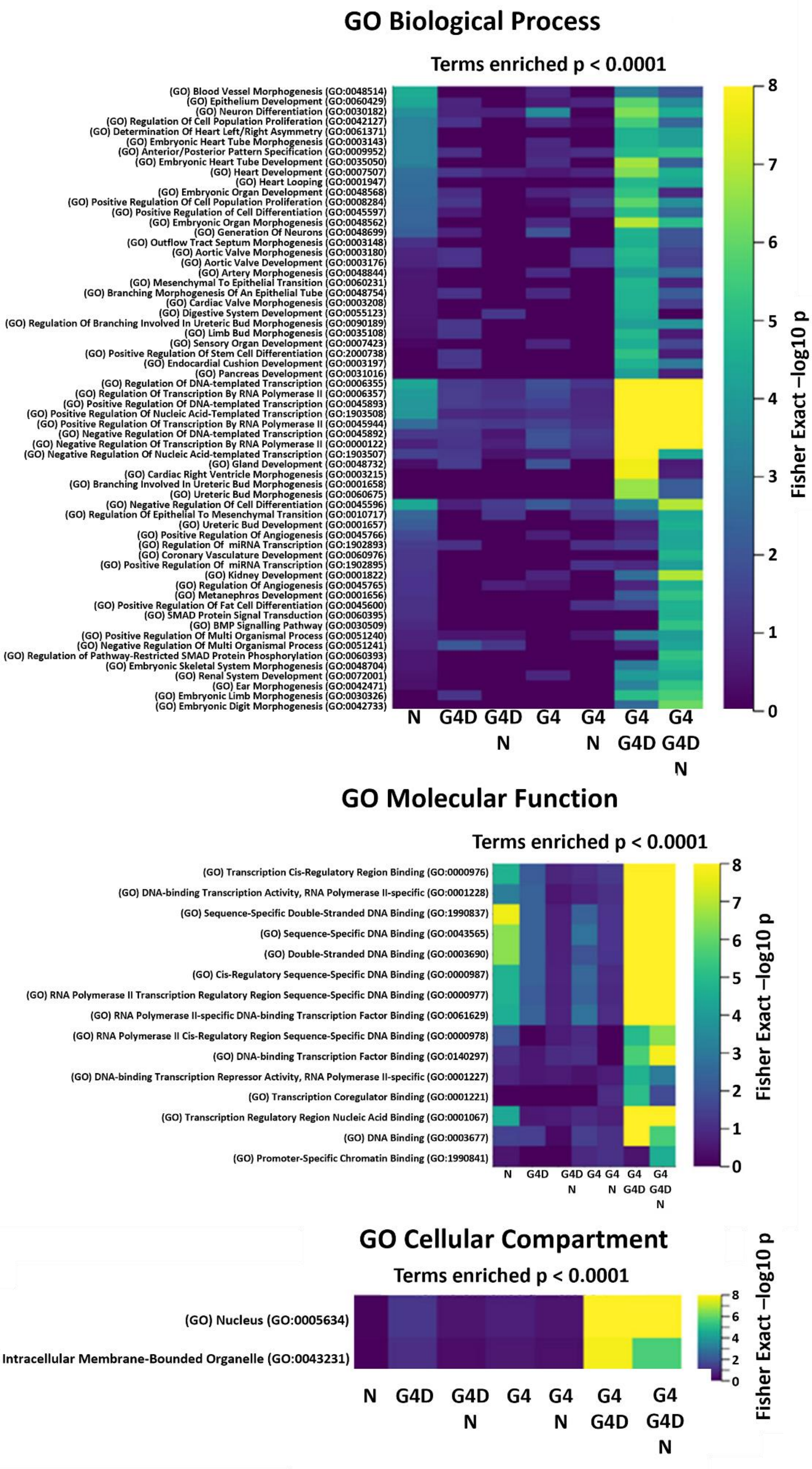

GO Biological Process
Terms enriched p < 0.0001
(GO) Blood Vessel Morphogenesis (GO:0048514)
(GO) Epithelium Development (GO:0060429)
(GO) Neuron Differentiation (GO:0030182)
(GO) Regulation Of Cell Population Proliferation (GO:0042127)
(GO) Determination Of Heart Left/Right Asymmetry (GO:0061371)
(GO) Embryonic Heart Tube Morphogenesis (GO:0003143)
(GO) Anterior/Posterior Pattern Specification (GO:0009952)
(GO) Embryonic Heart Tube Development (GO:0035050)
(GO) Heart Development (GO:0007507)
(GO) Heart Looping (GO:0001947)
(GO) Embryonic Organ Development (GO:0048568)
(GO) Positive Regulation Of Cell Population Proliferation (GO:0008284)
(GO) Positive Regulation of Cell Differentiation (GO:0045597)
(GO) Embryonic Organ Morphogenesis (GO:0048562)
(GO) Generation Of Neurons (GO:0048699)
(GO) Outflow Tract Septum Morphogenesis (GO:0003148)
(GO) Aortic Valve Morphogenesis (GO:0003180)
(GO) Aortic Valve Development (GO:0003176)
(GO) Artery Morphogenesis (GO:0048844)
(GO) Mesenchymal To Epithelial Transition (GO:0060231)
(GO) Branching Morphogenesis Of An Epithelial Tube (GO:0048754)
(GO) Cardiac Valve Morphogenesis (GO:0003208)
(GO) Digestive System Development (GO:0055123)
(GO) Regulation Of Branching Involved In Ureteric Bud Morphogenesis (GO:0090189)
(GO) Limb Bud Morphogenesis (GO:0035108)
(GO) Sensory Organ Development (GO:0007423)
(GO) Positive Regulation Of Stem Cell Differentiation (GO:2000738)
(GO) Endocardial Cushion Development (GO:0003197)
(GO) Pancreas Development (GO:0031016)
(GO) Regulation Of DNA-templated Transcription (GO:0006355)
(GO) Regulation Of Transcription By RNA Polymerase II (GO:0006357)
(GO) Positive Regulation Of DNA-templated Transcription (GO:0045893)
(GO) Positive Regulation Of Nucleic Acid-Templated Transcription (GO:1903508)
(GO) Positive Regulation Of Transcription By RNA Polymerase II (GO:0045944)
(GO) Negative Regulation Of DNA-templated Transcription (GO:0045892)
(GO) Negative Regulation Of Transcription By RNA Polymerase II (GO:0000122)
(GO) Negative Regulation Of Nucleic Acid-templated Transcription (GO:1903507)
(GO) Gland Development (GO:0048732)
(GO) Cardiac Right Ventricle Morphogenesis (GO:0003215)
(GO) Branching Involved In Ureteric Bud Morphogenesis (GO:0001658)
(GO) Ureteric Bud Morphogenesis (GO:0060675)
(GO) Negative Regulation Of Cell Differentiation (GO:0045596)
(GO) Regulation Of Epithelial To Mesenchymal Transition (GO:0010717)
(GO) Ureteric Bud Development (GO:0001657)
(GO) Positive Regulation Of Angiogenesis (GO:0045766)
(GO) Regulation Of miRNA Transcription (GO:1902893)
(GO) Coronary Vasculature Development (GO:0060976)
(GO) Positive Regulation Of miRNA Transcription (GO:1902895)
(GO) Kidney Development (GO:0001822)
(GO) Regulation Of Angiogenesis (GO:0045765)
(GO) Metanephros Development (GO:0001656)
(GO) Positive Regulation Of Fat Cell Differentiation (GO:0045600)
(GO) SMAD Protein Signal Transduction (GO:0060395)
(GO) BMP Signalling Pathway (GO:0030509)
(GO) Positive Regulation Of Multi Organismal Process (GO:0051240)
(GO) Negative Regulation Of Multi Organismal Process (GO:0051241)
(GO) Regulation of Pathway-Restricted SMAD Protein Phosphorylation (GO:0060393)
(GO) Embryonic Skeletal System Morphogenesis (GO:0048704)
(GO) Renal System Development (GO:0072001)
(GO) Ear Morphogenesis (GO:0042471)
(GO) Embryonic Limb Morphogenesis (GO:0030326)
(GO) Embryonic Digit Morphogenesis (GO:0042733)
N
G4D
G4D N
G4
G4 N
G4 G4D
G4 G4D N
8
7
6
5
4
3
2
1
0
Fisher Exact −log10 p
GO Molecular Function
Terms enriched p < 0.0001
(GO) Transcription Cis-Regulatory Region Binding (GO:0000976)
(GO) DNA-binding Transcription Activity, RNA Polymerase II-specific (GO:0001228)
(GO) Sequence-Specific Double-Stranded DNA Binding (GO:1990837)
(GO) Sequence-Specific DNA Binding (GO:0043565)
(GO) Double-Stranded DNA Binding (GO:0003690)
(GO) Cis-Regulatory Sequence-Specific DNA Binding (GO:0000987)
(GO) RNA Polymerase II Transcription Regulatory Region Sequence-Specific DNA Binding (GO:0000977)
(GO) RNA Polymerase II-specific DNA-binding Transcription Factor Binding (GO:0061629)
(GO) RNA Polymerase II Cis-Regulatory Region Sequence-Specific DNA Binding (GO:0000978)
(GO) DNA-binding Transcription Factor Binding (GO:0140297)
(GO) DNA-binding Transcription Repressor Activity, RNA Polymerase II-specific (GO:0001227)
(GO) Transcription Coregulator Binding (GO:0001221)
(GO) Transcription Regulatory Region Nucleic Acid Binding (GO:0001067)
(GO) DNA Binding (GO:0003677)
(GO) Promoter-Specific Chromatin Binding (GO:1990841)
N
G4D
G4D N
G4
G4 N
G4 G4D
G4 G4D N
8
7
6
5
4
3
2
1
0
Fisher Exact −log10 p
GO Cellular Compartment
Terms enriched p < 0.0001
(GO) Nucleus (GO:0005634)
(GO) Intracellular Membrane-Bounded Organelle (GO:0043231)
N
G4D
G4D N
G4
G4 N
G4 G4D
G4 G4D N
8
6
4
2
0
Fisher Exact −log10 p

Fig. S2. Gene ontology enrichments for genes differentially expressed during cardiogenesis induced by GATA4 (G4), GATA4+DKK1 (G4D) and Nodal5 (N) in *Xenopus* animal cap explants. Heatmap of gene ontology enrichments with FDR < 10-6 for sets of genes defined in upset plot in Figure 1b, heatmap as shown in Figure 1d, for Biological Process (top), Molecular Function (middle), and Cellular Compartment (bottom).

Biology Open • Supplementary information

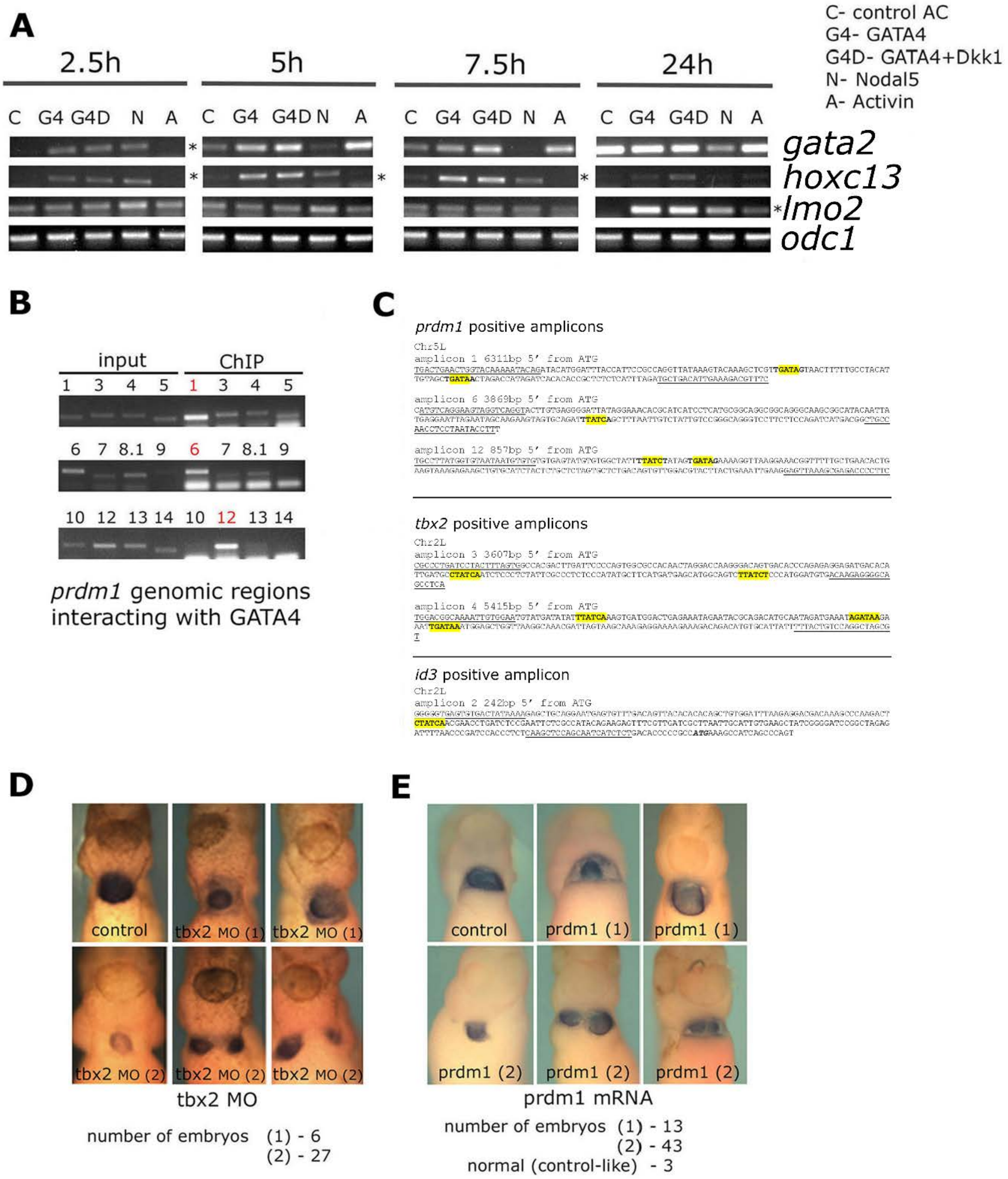

Fig. S3. **Additional *Xenopus* DEG validation data.** (A) Validation of further DEGs by RT-PCR. *gata2* is validated at 2.5h, but not at later time points when low dose of Activin, known to induce blood, induces the expression of this haematopoietic transcription factor. *hoxc13* is validated at all time points except 24h, whereas *lmo2* is only validated at 24h, when the level of expression induced by low dose of Activin is clearly lower than the level of expression induced by Nodal5, as well as G4 and G4D. *odc1* is used as a loading control. The same *odc1* data is shown in all panels where the same cDNA samples were used (Figs

Biology Open • Supplementary information

1E,G,H, 2A,D and S3A). (B) Three out of twelve tested *prdm1* genomic regions interact with GATA4. These are indicated by red numbers (1, 6, 12) in gel analysis of PCRs. The region 12 is the same as region 2 in Fig. 2F. (C) Sequences of *prdm1*, *tbx2* and *id3* ChIP-positive amplicons. Sequence used to derive PCR primers is underlined. Translation start ATG was used as starting point for numbering the sequences 5' from it. GATA sites are highlighted in yellow. (D, E) Additional images for in vivo analysis of *tbx2* and *prdm1* function. Ventral views of the head and heart region of st. 36/7 *Xenopus laevis* embryos processed for *myl7* whole mount in situ hybridisation to visualise the heart. Embryos injected with *tbx2* MO or *prdm1* mRNA were classified in 2 groups: (1) with milder phenotype (looping defects) and (2) with more severe phenotype (smaller linear heart tube, cardia bifida). The number of embryos in each group is indicated. Control embryos were uninjected. Anterior to the top.

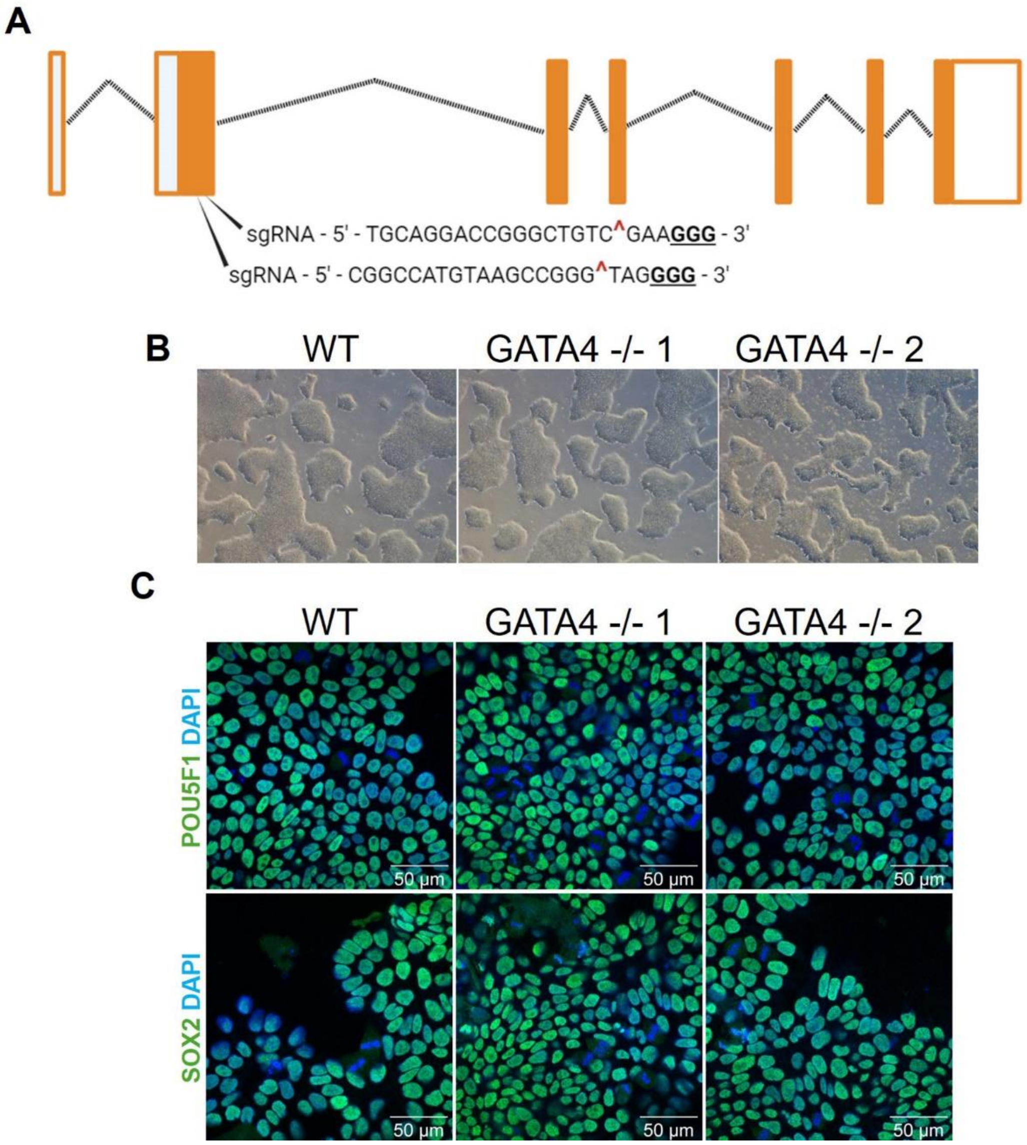

Fig. S4. Generation and pluripotency assessment of the of GATA4 -/- iPSC lines. (A) An overview of the human GATA4 gene locus. Exons are represented as boxes; coding regions are filled versus non-coding unfilled regions. Introns are represented by a dotted line. The CRISPR guides to edit the gene were targeted towards the end of the first coding exon and are labelled as sgRNA. The PAM for each is shown in bold and underlined. The cut site is indicated by a '^'. (B) Brightfield 4x images of WT and GATA4 null iPS cell colonies in culture. (C) Immunofluorescence for POU5F1 (green), and SOX2 (green) in WT and GATA4 null iPS cell colonies. DAPI (blue) was used to counterstain nuclei. Two independent lines with identical trans-heterozygous deletions (not shown) were created. In all figures samples labelled GATA4$^{-/-}$ were derived from the GATA4$^{-/-}$1 line.

Biology Open • Supplementary information

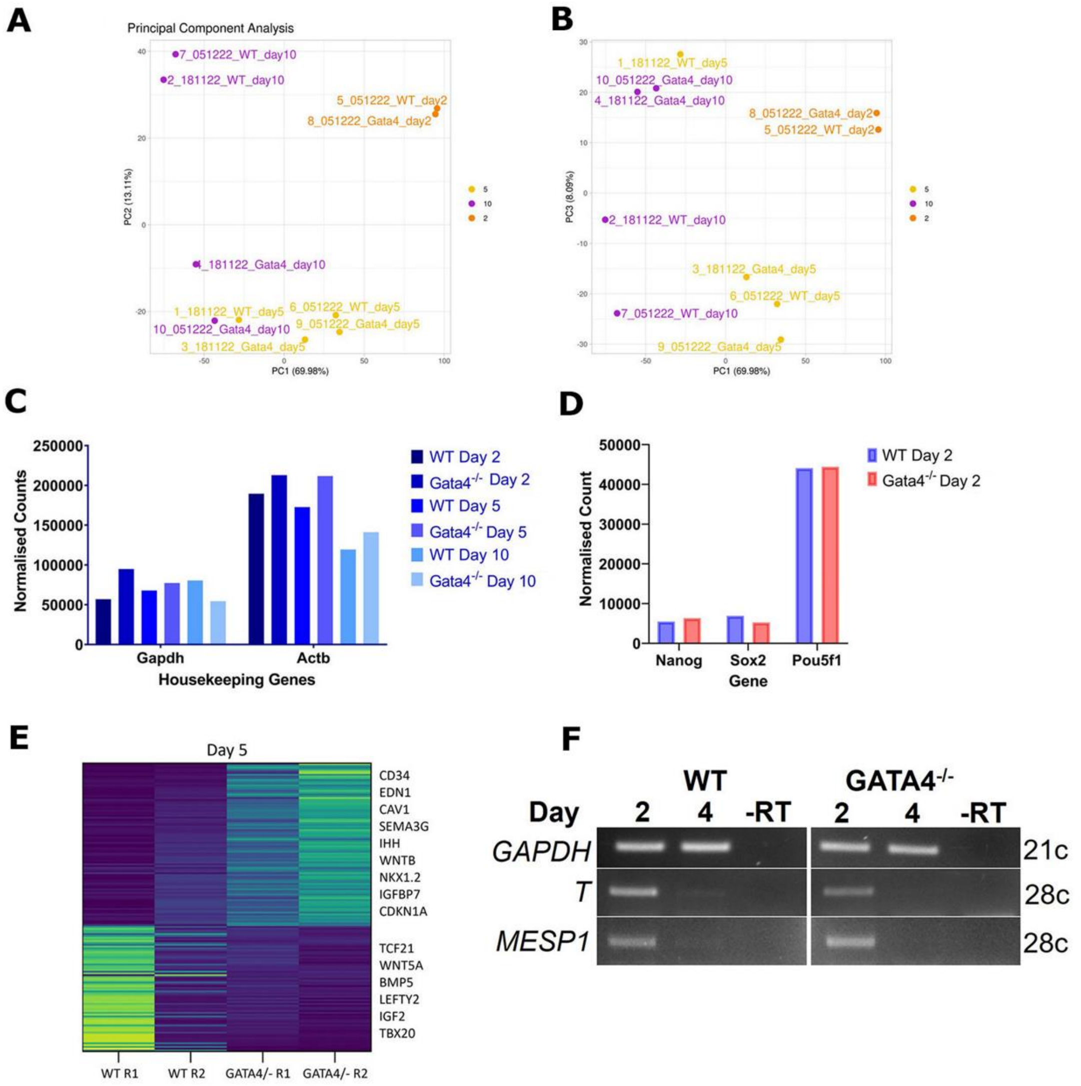

Fig. S5. Quality control of GATA4 -/- iPSC-CM RNA-seq data. (A-B) Principal component plots for WT and GATA4-/- samples at days 2 (n = 1), 5, and 10 of differentiation, n = 2. (C) The mean average of the normalised counts for housekeeping genes; *GAPDH* and *ACTB* are show for each time point. (D) A plot of normalised counts for WT and GATA4-/- samples at day 2 for pluripotency genes *NANOG*, *SOX2*, and *POU5F1,* n =1. (E) Heatmap of all genes found to be differentially expressed (DEGs) by a log2FC of 2 or more in the GATA4 -/- line at day 5, two repeats (R1, R2). Example genes are indicated on the right. (F) RT-PCR analysis showing comparable expression of *T* and *MESP1* in WT and GATA4-/- cells at days 2 and 4 of differentiation. *GAPDH* has been used as a loading control (n = 2).

Biology Open • Supplementary information

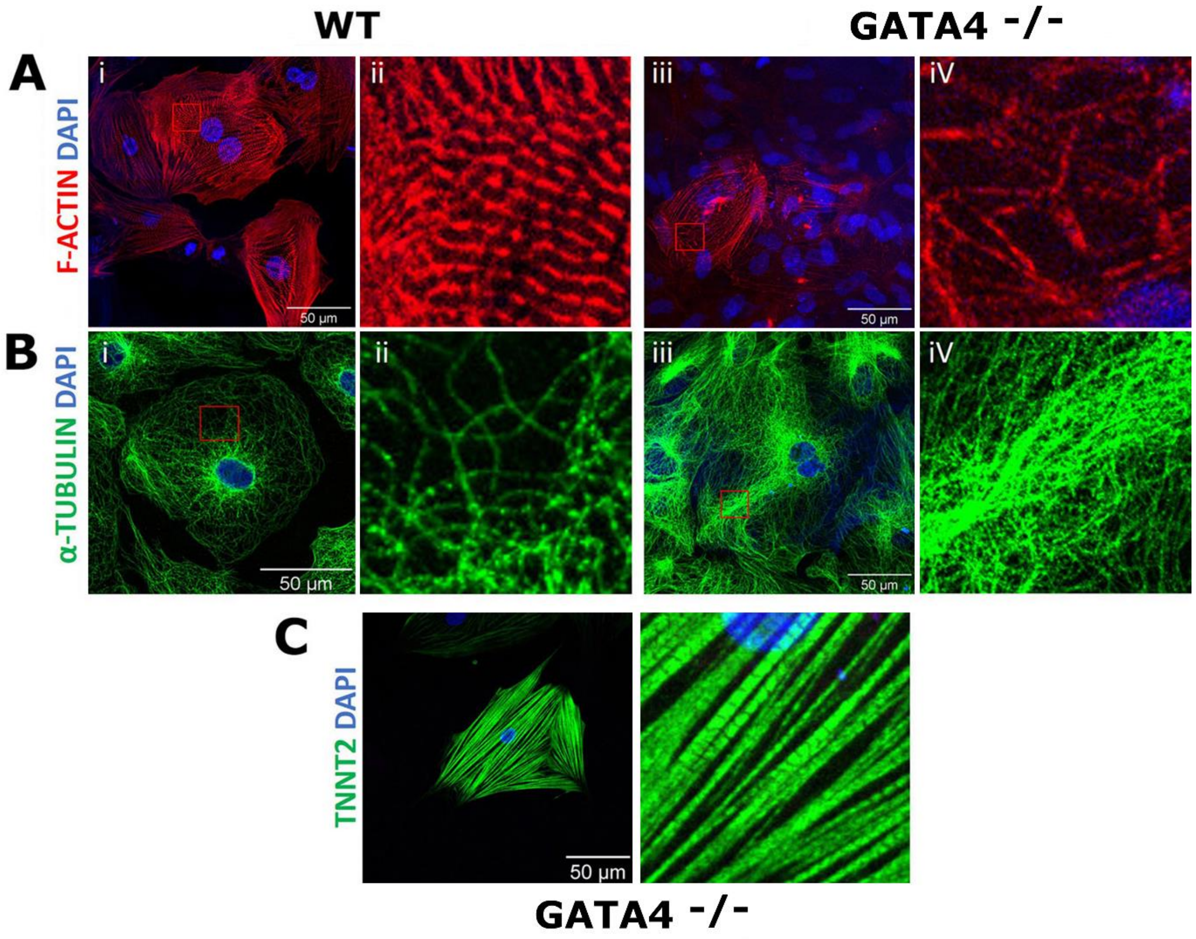

Fig. S6. Expression of F-ACTIN and α-TUBULIN in WT and GATA4 null cells.

IF staining was carried out on WT and GATA4-/- lines at day 32 and images taken at 40x with an oil immersion lens. Panel (A) shows staining for sarcomeric component Filamentous Actin (F-ACTIN). Panel (B) shows staining for non-sarcomeric cytoskeletal protein α-TUBULIN. Images labelled ii and iv show magnified cropped areas. In panel (C), a GATA4-/- cell with some striations is shown. Among the GATA4-/- cells analysed, this one had the mildest phenotype. See Fig. 4 legend for further details.

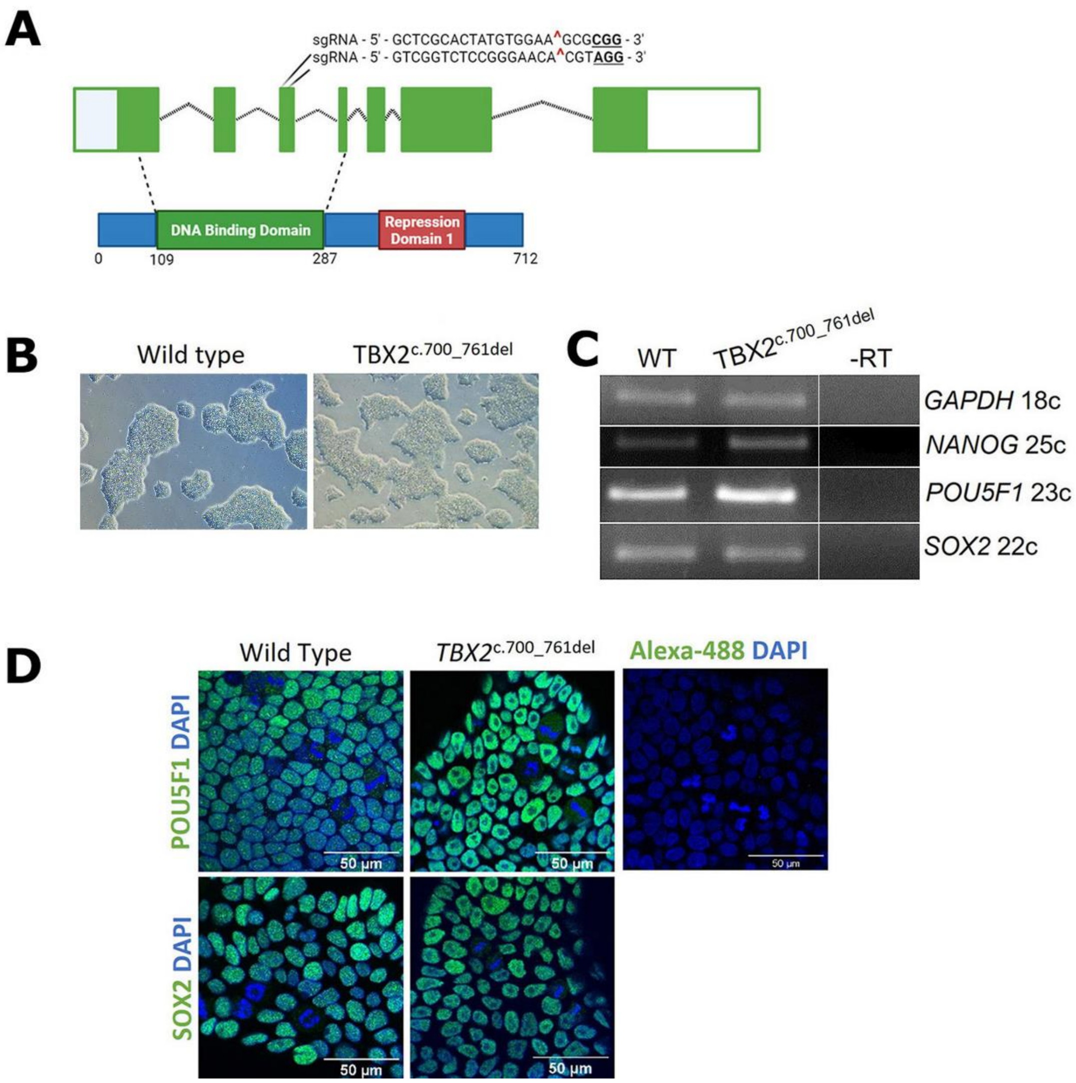

Fig. S7. Editing of the *TBX2* gene locus and pluripotency control of the mutant line. (A) A representation of the *TBX2* gene locus with exons shown as boxes. Coding regions are filled, and non-coding regions are empty. Introns are represented by a dotted line. The CRISPR guides used for editing were targeted to exon 3. The resulting mutation leads to the entirety of this exon being skipped. See Fig. S9 for more details. This is mapped onto the protein representation below to indicate the impact of this on the DNA binding domain. (B) Brightfield images showing WT and TBX2 c.700_761del iPSC colonies. (C) Expression of pluripotency factors NANOG, POU5F1, and SOX2 analysed by RT-PCR in WT and TBX2 mutant iPSCs. GAPDH is used as a normalisation control. (D) Immunofluorescent images of WT and TBX2 mutant iPSCs stained for POU5F1 and SOX2 (green), and counterstained with DAPI (blue). Taken at 63x with a confocal microscope.

Biology Open • Supplementary information

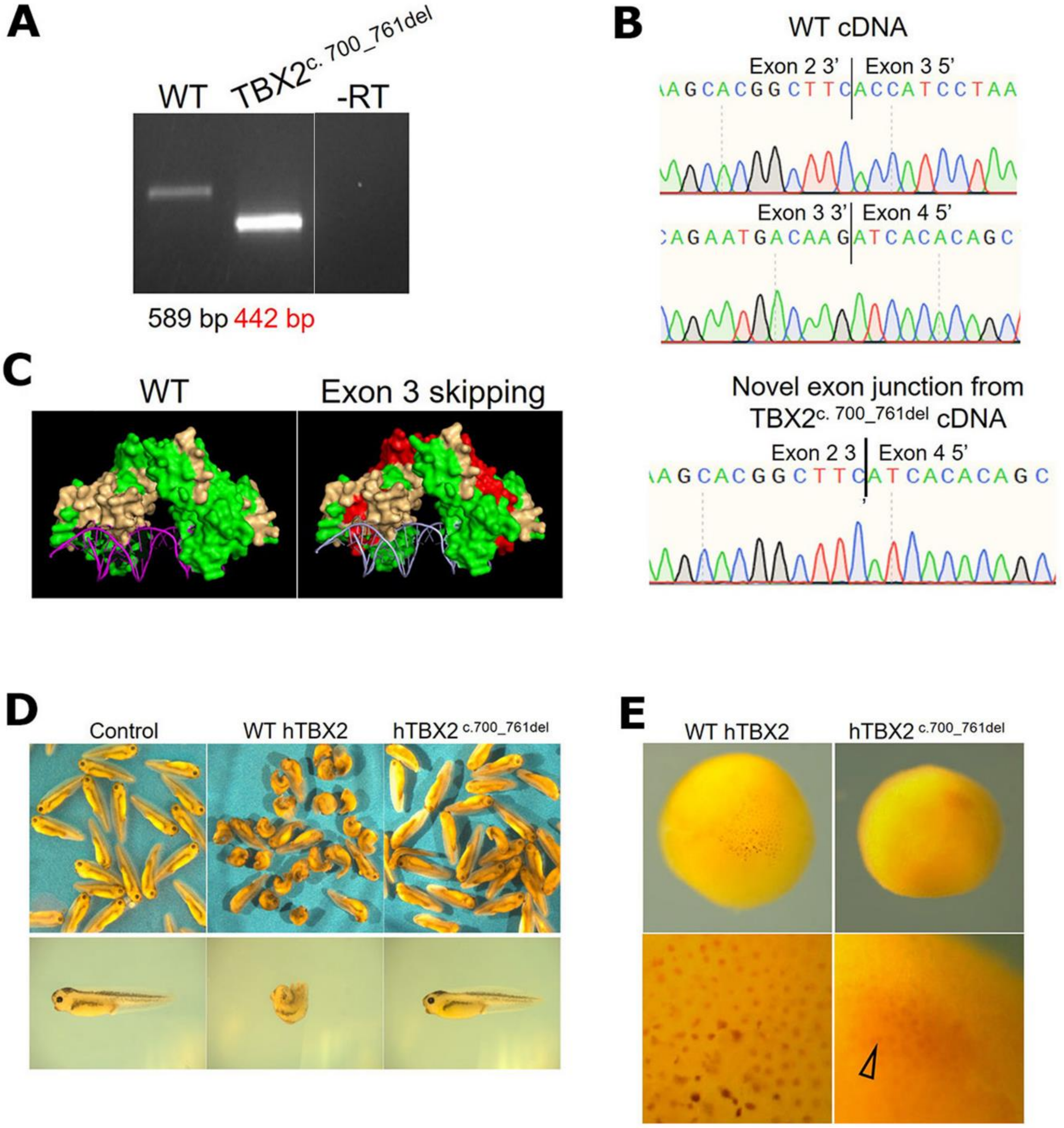

Fig. S8. The outcome of the TBX2 mutation. (A) RT-PCR analysis of day 10 samples shows based on the cDNA produced that the mRNA transcribed from the mutated gene locus misses ~150 bp. (B) Sanger sequencing trace files confirm 147bp deletion. The areas covering the 3' and 5' exon junctions from WT cDNA are shown alongside the new exon junction present in the TBX2 mutant that skips exon 3 (147 bp). (C) TBX3 is the closest relative of TBX2 with a 3D structure available. Here, the TBX3 DBD (PDB ID 1H6F) is shown binding to DNA as a homodimer. The regions absent in the TBX2 c.700_761del mutant line are shown in red. (D) Brightfield images showing stage 37/8 *Xenopus laevis* embryos, injected uniformly at the 2-4 cell stage with a WT h (human) TBX2 (n = 93) or hTBX2

Biology Open • Supplementary information

c.700_761del (n = 97) capped mRNA, in comparison to un-injected controls (n = 101). Combined numbers from 3 experiments. Single pictures displaying representative example embryos from each treatment group are shown below. The embryos injected with WT TBX2 mRNA show strong gastrulation defects, but those injected with mutant TBX2 mRNA are indistinguishable from controls. (I) Immunohistochemical staining in stage 9.5 embryos using an anti-HA antibody to detect HA tagged WT and mutant hTBX2 proteins. The WT but not mutant protein is readily detected in the nuclei. Taken together, the data shown here and in Fig. 6A indicate that the deletion of aa. 700-761 removes part of DNA binding domain and leads to destabilisation of TBX2 protein, resulting in a functional null mutation, at least when expressed in *Xenopus* embryos. Indirect assessment of TBX2 mutant protein in *Xenopus* embryos was undertaken as commercially available antibodies against human TBX2 showed low sensitivity.

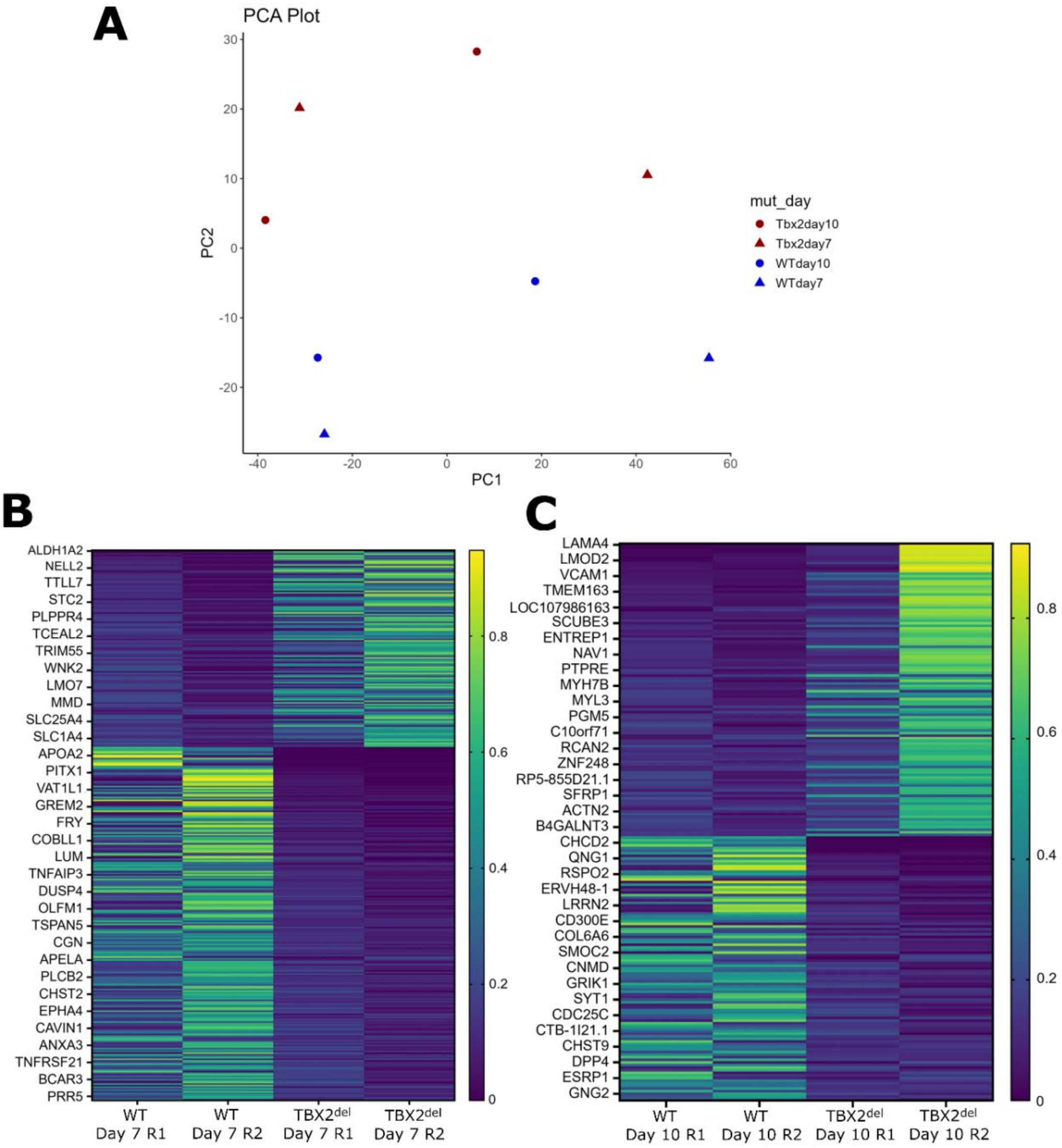

Fig. S9. Quality control of TBX2 -/- iPSC-CM RNA-seq data and selected up- and down-regulated genes. (A) PCA plot showing WT vs TBX2 mutant samples at day 7 and day 10 of differentiation. (B) A heatmap showing DEGs at day 7 in the TBX2 mutant line and (C) at day 10. Whilst the upregulated genes and GO terms are more prominent (p values in the 10^{-11}-10^{-20} range; Fig. 6, Supplementary Table 2), the downregulated genes and GO terms (p values in the 10^{-5}-10^{-9} range; Supplementary Table 2), include ECM genes such as COL6A6, RSPO2 and SMOC2 (panel C) and GO terms such as 'ECM', 'extracellular region' and 'collagen-containing extracellular matrix' (Supplementary Table 2).

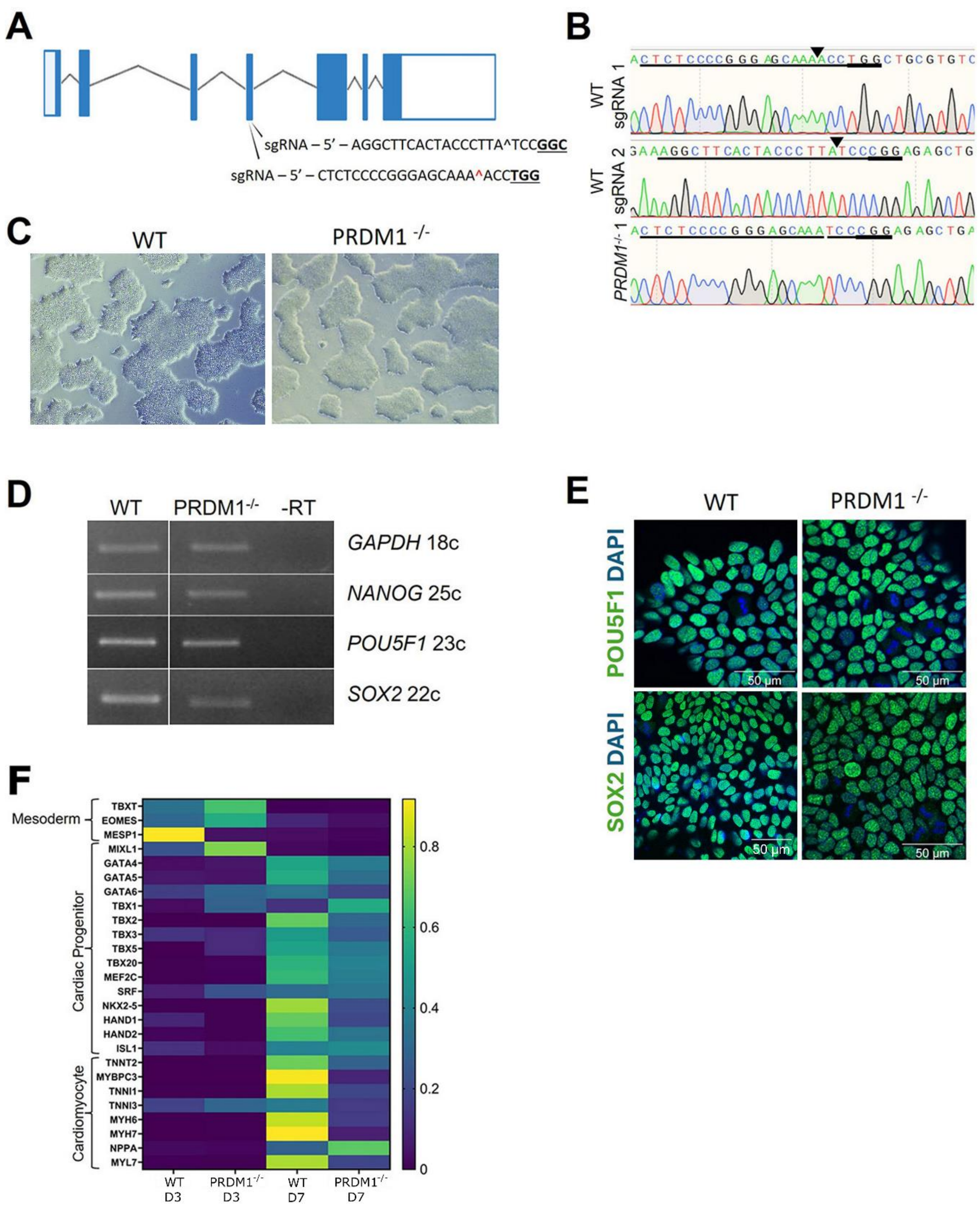

Fig. S10. Generation and pluripotency assessment of the PRDM1 -/- iPSC line. (A) The PRDM1 gene locus with exons depicted as boxes and coding regions of these as filled boxes. Introns are represented as dotted lines. The guide RNAs used to knockout PRDM1 were targeted to exon 4 as this is shared amongst all PRDM1 isoforms. The PAM sequence is shown in bold and underlined. The cut site is indicated by '^'. (B) Sanger sequencing trace files showing the locus before and after editing. Guide RNAs have been underlined with PAM

Biology Open • Supplementary information

sites underlined in bold. Cut sites indicated with a black arrow. (C) Brightfield images showing WT and PRDM1-/- iPS cell colonies. (D) RT-PCR showing the expression of NANOG, POU5F1, and SOX2 expression in WT and PRDM1-/- 1 iPSCs. GAPDH was used as a normalisation control. (E) Immunofluorescence images showing POU5F1 (green), and SOX2 (green) expression in WT and PRDM1-/- cells with DAPI (blue) used to counterstain nuclei. This demonstrates localisation of these TFs to the nucleus in non-dividing cells. (F) A heatmap generated from RNA-seq data showing the expression of mesoderm, cardiac progenitor and cardiomyocyte markers as days 3 and 7 of differentiation in the WT and PRDM1 -/- line.

Table S1. Gene set enrichment analysis for the overlap in differentially expressed genes in *Xenopus* explants treated with Gata4 (G4), Gata4 and Dkk1 (G4D), or Nodal (N).

Available for download at
https://journals.biologists.com/bio/article-lookup/doi/10.1242/bio.062553#supplementary-data

Table S2. Gene set enrichment analysis for the human iPSC lines at the days indicated.

Available for download at
https://journals.biologists.com/bio/article-lookup/doi/10.1242/bio.062553#supplementary-data

Table S3. Normalised count from RNA-seq analysis of the WT Rebl Pat parental human iPS cell line and its mutant derivatives at different days of iPSC-CM differentiation.

Available for download at
https://journals.biologists.com/bio/article-lookup/doi/10.1242/bio.062553#supplementary-data

Table S4. Information on antibodies, sgRNAs and primers used in this study.

Available for download at
https://journals.biologists.com/bio/article-lookup/doi/10.1242/bio.062553#supplementary-data

Table S5. Karyotyping data for the parental Rebl Pat iPS cell line and its edited derivatives

Available for download at
https://journals.biologists.com/bio/article-lookup/doi/10.1242/bio.062553#supplementary-data
