# Supplementary material for: Conserved roles of GATA4 and its target gene TBX2 in regulation of human cardiogenesis
Source: Biol Open. 2026 Jun 15;15(6):bio062553. doi: 10.1242/bio.062553 (PMC13312917; doi:10.1242/bio.062553)
Supplement: Supplementary file 1 [file biolopen-15-062553_review_history.pdf]

Conserved roles of GATA4 and its target gene TBX2 in regulation of human cardiogenesis

Nicola Graham, Pavel Kirilenko, Ilya Patrushev, Ewan D. Fowler, Peter Kille, Michael Gilchrist, Nick D.L. Owens and Branko Latinkic

10.1242/bio.062553

Editor: Tristan Rodríguez

Review timeline

Original submission:	3 March 2026
Editorial decision:	12 March 2026
First revision received:	24 April 2026
Accepted:	28 April 2026

Original submission

First decision letter

MS ID#: bio.062553

MS Title: Conserved roles of GATA4 and its target gene TBX2 in regulation of human cardiogenesis

Authors: Nicola Graham; Pavel Kirilenko; Ilya Patrushev; Ewan D. Fowler; Peter Kille; Michael Gilchrist; Nick D.L. Owens; Branko Latinkic

I hope all is well. I have now reached a decision on the above manuscript.

The reviewer reports are shown at the bottom of this email.

As you will see, the reviewers raised a number of substantial criticisms that prevent me from accepting the paper at this stage.

They suggest, however, that a revised version would prove acceptable, if you can address their concerns. If you think that you can deal satisfactorily with the criticisms on revision, I would be pleased to see a revised manuscript. I do not think that there is a requirement for additional experimentation, but if the points raised cannot be addressed without experimentation, I would suggest modifying some of the claims of the manuscript so that they are more circumspect. However, should the requested experimental results be available then please provide these in the revised manuscript.

At this stage, we also ask you to ensure your manuscript complies with our formatting guidelines. Provided you are able to fully address the referees' comments, we are positive about publication of your paper (we accept over 95% of revision submissions) and therefore hope you won't mind any extra work involved in reformatting your manuscript at this point.

Please upload both a 'clean' version of your Word file, along with a highlighted version clearly showing where you have made changes in the revised manuscript. Please avoid using 'Track changes' in Word files as these are lost in PDF conversion.

© 2026. Published by The Company of Biologists under the terms of the Creative Commons Attribution License (https://creativecommons.org/licenses/by/4.0/).

I should be grateful if you would also provide a point-by-point response detailing how you have dealt with the points raised by the reviewers in the 'Response to Reviewers' box. Please attend to all of the reviewers' comments. If you do not agree with any of their criticisms or suggestions please explain clearly why this is so.

Reviewer 1

Comments for the author

In this manuscript, the authors investigate the role of GATA4 and Nodal as cardiogenic factors and aim to identify components of the cardiac gene regulatory networks (GRNs) controlled by these signals. Because both GATA4 and Nodal are known to induce cardiomyocyte differentiation, the authors use these cardiogenic triggers to identify and characterize co-regulated differentially expressed genes (DEGs) that may function as components of cardiac GRNs. A subset of the identified DEGs is validated as GATA4 targets both in vivo and in explant assays. Functional interference with two of these targets, tbx2 and prdm1, is shown to affect heart development in Xenopus embryos. The broader relevance of these findings is supported by experiments in human iPSC-derived cardiomyocytes, demonstrating essential roles for GATA4 and its downstream target TBX2 during cardiomyocyte differentiation.

The study begins by identifying candidate GATA4 targets using a previously established Xenopus explant culture system. The DEGs identified through this screening are subsequently validated by RT-PCR and computational analyses. Among these genes, TBX2 and PRDM1 are proposed to be directly regulated by GATA4.

The authors then extend their analysis to human iPSCs, where they show that GATA4 knockout iPSCs exhibit impaired differentiation into cardiomyocytes, and that GATA4 regulates TBX2 and PRDM1 expression in this system. Figure 4 is particularly well presented, and I like it a lot. Finally, the authors demonstrate that knockout of TBX2 or PRDM1 in human iPSCs also results in differentiation defects, although these phenotypes appear less severe than those observed in GATA4-/- cells.

The manuscript contains a very large amount of experimental data, and the authors have generally taken care to validate their findings using multiple approaches (e.g., RT-PCR, western blotting, and immunostaining). Although RT-PCR is sometimes considered an older method, it remains a valid and informative approach when carefully performed and appropriately controlled.

The manuscript would nevertheless benefit from some condensation, as certain sections, particularly the Introduction and Discussion are somewhat longer than necessary.

I have several minor comments:

1. Figure citation order: Figures are not always cited in the correct sequence, either within individual figures (less important) or across the manuscript. For example, Figure 5H is mentioned before Figure 4. The latter could be easily corrected.

2. Figure 2G clarity: Figure 2G is somewhat confusing. I suggest clearly indicating the probe used for the in situ hybridization and explicitly specifying the experimental manipulation (e.g., TBX2 morpholino or PRDM1 overexpression) within the figure.

3. Experimental sample sizes: The number of explants used in the experiments is not reported (e.g., in RT-PCR experiments or RNA-seq analyses). This information should be included in the Materials and Methods section. Similarly, for the iPSC experiments, the number of clones analyzed and the number of biological replicates should be clearly specified for each experiments.

Reviewer 2

Comments for the author

© 2026. Published by The Company of Biologists under the terms of the Creative Commons Attribution License (https://creativecommons.org/licenses/by/4.0/).

The manuscript by Graham et al. uses a strong cross-species strategy (Xenopus gain of function screen with in vivo validation, followed by human iPSC-CM genetics) to expand the GATA4-regulated cardiogenic network, with a focus on TBX2 and PRDM1. Several datasets are compelling, particularly the marked differentiation failure in GATA4-null cells and the TBX2 mutant iPSC-CM phenotype. However, key controls, replicate depth and tuning down a few overstated claims need attention to ensure the conclusions match the evidence and the work is readily reproducible. Please see major and minor points below.

Major points

- The authors activate GATA4-GR with DEX, yet the stated RNA-seq control is uninjected animal caps (Fig 1A). DEX exposure and injection stress can alter transcription. The authors should including DEX-treated uninjected caps or injected but not activated controls or justify why these are unnecessary. Without these, a subset of DEGs may not be cardiogenesis-specific.

- Several key RNA-seq comparisons are underpowered: GATA4 day 2 is n=1 (Fig S5) and PRDM1 day 7 is n=1 (Fig 7E). Single replicate RNA-seq falls short of being suitable for robust differential expression or GO inference. Therefore, the authors should increase biological replicates for these datasets and state clearly for every main figure what n represents (independent differentiations vs technical repeats?).

- The approach re minimum padj across timepoints and DEG definition "irrespective of timepoint" causes a problem by inflating significance across repeated testing and can overcall transient or more noisy effects. The authors should either use a time course model or a more conservative rule and report effect sizes per timepoint.

- The authors should clarify the xenopus sampling scheme and reconcile timepoints as in the Results section, the authors describe 2.5h, 5h and 7.5h post-excision/activation but other sections refer to 24h. If 24h is included in sequencing/DE testing, state this explicitly and present it consistently. If it is only for RT-PCR validation, state that clearly.

- The ChIP-PCR data (HA-GATA4 overexpression) are suggestive but these need stronger specificity controls to support "direct binding" language. The authors should include IgG/no-tag controls, negative genomic regions or provide quantitative ChIP-qPCR with those controls and replicate quantification for the key loci presented.

- The statement that GATA4 loss promotes smooth muscle/fibroblast/myofibroblast programmes is plausible but bulk RNA-seq marker shifts can simply reflect altered within-cell composition rather than cell fate shifts. The authors could add cell type validation via flow or immunostaining with quantification for CM, endothelial and mesenchymal markers to make these claims stronger.

- NPPA/NPPB induction and increased cell size support a hypertrophic-like phenotype but iPSC-CM maturity, density and pacing state can influence these readouts. I would therefore suggest the authors to frame conclusions as "hypertrophic-like features consistent with maladaptive remodelling" unless you add more direct functional measures (e.g, contractility and electrophysiology).

Minor points

- Figure legends state activation with 1 mM dex. This reads unusually high. If correct, justify but if a unit error, correct throughout.

- Where outcomes are proportions across wells or cells, ensure the statistical test matches the experimental unit and clarify whether n is differentiations, wells, images or cells, etc.
- Please revise Fig 7 legend to correct errors in text (G and G?).

Reviewer's Responses to Questions

Experimental quality

© 2026. Published by The Company of Biologists under the terms of the Creative Commons Attribution License (https://creativecommons.org/licenses/by/4.0/).

Does each figure have the proper controls?
If 'No', please indicate reasons in Comments for Author box below.
Reviewer #1:
- Yes

Reviewer #2:
- No

Were the data analyzed using appropriate statistical tests?
If 'No', please indicate reasons in Comments for Author box below.
Reviewer #1:
- Yes

Reviewer #2:
- Yes

Reproducibility
Were experiments performed using adequate number of biological replicates?
If 'No', please indicate reasons in Comments for Author box below.
Reviewer #1:
- Yes

Reviewer #2:
- No

Does the methods section provide sufficient detail to permit reproducibility?
If 'No', please indicate reasons in Comments for Author box below.
Reviewer #1:
- Yes

Reviewer #2:
- Yes

Completeness
Are the manuscript's conclusions supported by the data?
If 'No', please indicate reasons in Comments for Author box below.
Reviewer #1:
- Yes

Reviewer #2:
- No

Scholarship
Do the authors cite and discuss the merits of data that would argue for and against their conclusion?
If 'No', please indicate reasons in Comments for Author box below.
Reviewer #1:
- Yes

Reviewer #2:
- Yes

© 2026. Published by The Company of Biologists under the terms of the Creative Commons Attribution License (https://creativecommons.org/licenses/by/4.0/).

Does the manuscript title & abstract accurately reflect the contents of the manuscript, without hyperbole?
If 'No', please indicate reasons in Comments for Author box below.
Reviewer #1:

- Yes

Reviewer #2:

- Yes

First revision

Author response to reviewers' comments

We are grateful to the reviewers for their constructive criticism which has helped to improve the manuscript. All comments have been addressed.

Reviewer 1: In this manuscript, the authors investigate the role of GATA4 and Nodal as cardiogenic factors and aim to identify components of the cardiac gene regulatory networks (GRNs) controlled by these signals. Because both GATA4 and Nodal are known to induce cardiomyocyte differentiation, the authors use these cardiogenic triggers to identify and characterize co-regulated differentially expressed genes (DEGs) that may function as components of cardiac GRNs. A subset of the identified DEGs is validated as GATA4 targets both in vivo and in explant assays. Functional interference with two of these targets, tbx2 and prdm1, is shown to affect heart development in Xenopus embryos. The broader relevance of these findings is supported by experiments in human iPSC-derived cardiomyocytes, demonstrating essential roles for GATA4 and its downstream target TBX2 during cardiomyocyte differentiation.

The study begins by identifying candidate GATA4 targets using a previously established Xenopus explant culture system. The DEGs identified through this screening are subsequently validated by RT-PCR and computational analyses. Among these genes, TBX2 and PRDM1 are proposed to be directly regulated by GATA4.

The authors then extend their analysis to human iPSCs, where they show that GATA4 knockout iPSCs exhibit impaired differentiation into cardiomyocytes, and that GATA4 regulates TBX2 and PRDM1 expression in this system. Figure 4 is particularly well presented, and I like it a lot. Finally, the authors demonstrate that knockout of TBX2 or PRDM1 in human iPSCs also results in differentiation defects, although these phenotypes appear less severe than those observed in GATA4-/- cells.

The manuscript contains a very large amount of experimental data, and the authors have generally taken care to validate their findings using multiple approaches (e.g., RT-PCR, western bloting, and immunostaining). Although RT-PCR is sometimes considered an older method, it remains a valid and informative approach when carefully performed and appropriately controlled.

The manuscript would nevertheless benefit from some condensation, as certain sections, particularly the Introduction and Discussion are somewhat longer than necessary.

We have condensed the manuscript as much as we were able to, without affecting its clarity. In the highlighted version of the revised manuscript the deleted text is in red. The text added to address other comments of the reviewers is highlighted in yellow. There is more red- than yellow-highlighted text, indicating that the manuscript has been condensed.

I have several minor comments:

© 2026. Published by The Company of Biologists under the terms of the Creative Commons Attribution License (https://creativecommons.org/licenses/by/4.0/).

1. Figure citation order: Figures are not always cited in the correct sequence, either within individual figures (less important) or across the manuscript. For example, Figure 5H is mentioned before Figure 4. The later could be easily corrected.

We have corrected this oversight and thank the reviewer for alerting us to it.

2. Figure 2G clarity: Figure 2G is somewhat confusing. I suggest clearly indicating the probe used for the in situ hybridization and explicitly specifying the experimental manipulation (e.g., TBX2 morpholino or PRDM1 overexpression) within the figure.

We have amended the figure by adding "myl7 (heart)" to indicate the probe used to visualise the heart. The samples injected with tbx2 morpholino are labelled "tbx2 MO" and those injected with prdm1 mRNA are labelled "prdm1". This is clearly stated in the figure legend.

3. Experimental sample sizes: The number of explants used in the experiments is not reported (e.g., in RT-PCR experiments or RNA-seq analyses). This information should be included in the Materials and Methods section. Similarly, for the iPSC experiments, the number of clones analyzed and the number of biological replicates should be clearly specified for each experiments.

The number of explants used has now been included in the Methods section.
For the iPSC-CM experiments, n refers to RNA samples derived from independent experiments. For each sample, three wells per plate were pooled to minimize well-to-well variation. In the GATA4-null dataset, day 2 samples have n = 1, while all other time points have an n of 2. All RNA-seq samples relating to the TBX2 lines have an n of 2, and all PRDM1 timepoints have an n = 1.

Reviewer 2: The manuscript by Graham et al. uses a strong cross-species strategy (Xenopus gain of function screen with in vivo validation, followed by human iPSC-CM genetics) to expand the GATA4-regulated cardiogenic network, with a focus on TBX2 and PRDM1. Several datasets are compelling, particularly the marked differentiation failure in GATA4-null cells and the TBX2 mutant iPSC-CM phenotype. However, key controls, replicate depth and tuning down a few overstated claims need atention to ensure the conclusions match the evidence and the work is readily reproducible. Please see major and minor points below.

Major points

- The authors activate GATA4-GR with DEX, yet the stated RNA-seq control is uninjected animal caps (Fig 1A). DEX exposure and injection stress can alter transcription. The authors should including DEX-treated uninjected caps or injected but not activated controls or justify why these are unnecessary. Without these, a subset of DEGs may not be cardiogenesis- specific.

We and others have extensively used DEX treatment of Xenopus animal cap explants to activate various GR fusion constructs and have not observed DEX-specific effects. For example, a 1997 study by Tada et al (10.1242/dev.124.11.2225) documents this. The DEX alone treatment was omitted in RNA-seq samples which involved dissection of ~300 explants in a single experiment, to reduce the number of non-essential samples. In most validation experiments we injected the non-inducible version of GATA4 and observed identical results (data not shown). In addition, our definition of cardiogenic DEGs relied on their co-regulation by all 3 cardiogenic conditions including Nodal, which did not include DEX treatment. Taken together, our results and published evidence suggest that our findings are not affected by DEX-specific effects.

- Several key RNA-seq comparisons are underpowered: GATA4 day 2 is n=1 (Fig S5) and PRDM1 day 7 is n=1 (Fig 7E). Single replicate RNA-seq falls short of being suitable for robust differential expression or GO inference. Therefore, the authors should increase biological replicates for these datasets and state clearly for every main figure what n represents (independent differentiations vs technical repeats?).

© 2026. Published by The Company of Biologists under the terms of the Creative Commons Attribution License (https://creativecommons.org/licenses/by/4.0/).

We acknowledge the reviewer's concern that some of the RNA-seq datasets are underpowered and wish to clarify that all iPSC-CM RNA-seq samples shown are derived from biological replicates, for the WT vs. GATA4 comparison at day 2, n = 1, whereas for the other time points n = 2 in all cases. The day 2 RNA-seq findings are supported by RT-PCR data (n = 2), for marker genes T and MESP1. These genes are well-established markers of mesodermal and cardiac progenitor development at this stage and support our conclusion that these processed are not perturbed in the GATA4 null line, as expected from very low expression of GATA4 at this time point. The day 2 sample can be viewed as a control which is not expected to offer new insights, and this appears to be confirmed by a single repeat RNA-seq experiment and RT-PCR for T and MESP1. However, we recognise the limitations of our data and have revised the text to reflect this more clearly.
Regarding the PRDM1 RNA-seq data, the number of replicates is just 1 per time point. This is stated in the manuscript, and we emphasise that conclusions drawn from these data are cautiously interpreted with this limitation in mind. Nevertheless, we believe that sharing the dataset and clearly stating its limitations offers useful biological insight.

- The approach re minimum padj across timepoints and DEG definition "irrespective of timepoint" causes a problem by inflating significance across repeated testing and can overcall transient or more noisy effects. The authors should either use a time course model or a more conservative rule and report effect sizes per timepoint.

The reviewer is correct in that DEGs will contain false positives, however, given that the purpose of the RNA-seq experiment was enrichment of DEGs and hypothesis generation, we opted for this analysis given the constraints of the experimental design. We took care to analyse individual timepoints and confirm that each timepoint gave a concordant view (see Fig S1B-F). We experimented with alternative conditions and found that either a time series analysis or a condition sharing information between timepoints gave no substantial benefit whilst introducing other biases. We therefore opted for the condition, knowing that we are both underpowered and likely have false positives. However, we again highlight that despite these limitations, the pattern of transcriptomic time course experiment provided robust hypotheses and sets of candidate DEGs which we then validated by independent means.

- The authors should clarify the xenopus sampling scheme and reconcile timepoints as in the Results section, the authors describe 2.5h, 5h and 7.5h post-excision/activation but other sections refer to 24h. If 24h is included in sequencing/DE testing, state this explicitly and present it consistently. If it is only for RT-PCR validation, state that clearly.

We thank the reviewer for pointing this inconsistency in provided information, which we have corrected. The 24h time point was included in the RNA-seq experiment.

- The ChIP-PCR data (HA-GATA4 overexpression) are suggestive but these need stronger specificity controls to support "direct binding" language. The authors should include IgG/no- tag controls, negative genomic regions or provide quantitative ChIP-qPCR with those controls and replicate quantification for the key loci presented.

In the Results (p. 7) we have removed the word 'directly' in "We next asked if GATA4 directly regulates *id3, prdm1 and tbx2...".*
In the Discussion we stated that "...prdm1 and tbx2 [whose] expression in Xenopus embryos is regulated by GATA4, possibly directly." We are not stating that regulation is through direct binding, but this is possible and is consistent with association of exogenous GATA4 with prdm1 and tbx2 regulatory regions in chromatin (Fig. 2 and Fig. S3). In the Results section, we stated: "the minority of tested sites were found to associate with GATA4 (Fig. 2C, F and Fig S3B-C), suggesting that GATA4 is directly and specifically regulating their expression". Thisaddresses the reviewer's comment regarding negative genomic loci. A clear limitation of the approach based on exogenous GATA4 we used is that it can show what are possible chromatin associations of GATA4 but not what is the actual chromatin association of the endogenous GATA4. Thus, we stated that our data is suggesting but not demonstrating regulation through direct binding.

© 2026. Published by The Company of Biologists under the terms of the Creative Commons Attribution License (https://creativecommons.org/licenses/by/4.0/).

- The statement that GATA4 loss promotes smooth muscle/fibroblast/myofibroblast programmes is plausible but bulk RNA-seq marker shitis can simply reflect altered within-cell composition rather than cell fate shitis. The authors could add cell type validation via flow or immunostaining with quantification for CM, endothelial and mesenchymal markers to make these claims stronger.

We agree that our data does not allow us to conclude that a cell fate switch has occurred. As we stated in the manuscript, this is the key limitation of the current work. The most direct approach to resolve it in future studies would be to establish developmental trajectories of wt vs. GATA4 null cells using scRNA-seq. Establishing the onset of divergence of mutant cell transcriptional profiles as well as their differentiated identities will set the scene for investigation of the mechanisms through which GATA4 acts to regulate this process.

- NPPA/NPPB induction and increased cell size support a hypertrophic-like phenotype but iPSC-CM maturity, density and pacing state can influence these readouts. I would therefore suggest the authors to frame conclusions as "hypertrophic-like features consistent with maladaptive remodelling" unless you add more direct functional measures (e.g, contractility and electrophysiology).

We agree and thank the reviewer for this helpful suggestion. The manuscript has been revised accordingly.

Minor points

- Figure legends state activation with 1 mM dex. This reads unusually high. If correct, justify but if a unit error, correct throughout.

Thank you, the Greek μ was lost in manuscript reformatting. This has been corrected.

- Where outcomes are proportions across wells or cells, ensure the statistical test matches the experimental unit and clarify whether n is differentiations, wells, images or cells, etc.

Additional information clarifying this point is provided in the Methods section and in figure legends.

- Please revise Fig 7 legend to correct errors in text (G and G?).

We thank the reviewer for noting this omission, which has been corrected (a description of panel H was added).

<u>Second decision letter</u>

MS ID#: bio.062553R1

MS Title: Conserved roles of GATA4 and its target gene TBX2 in regulation of human cardiogenesis

Authors: Nicola Graham; Pavel Kirilenko; Ilya Patrushev; Ewan D. Fowler; Peter Kille; Michael Gilchrist; Nick D.L. Owens; Branko Latinkic

I am happy to tell you that your manuscript has been accepted for publication in Biology Open, pending our standard publication integrity checks. It was accepted on 28th April 2026.

© 2026. Published by The Company of Biologists under the terms of the Creative Commons Attribution License (https://creativecommons.org/licenses/by/4.0/).
